# Poly-L-Lactic Acid Fillers Improved Dermal Collagen Synthesis by Modulating M2 Macrophage Polarization in Aged Animal Skin

**DOI:** 10.3390/cells12091320

**Published:** 2023-05-05

**Authors:** Seyeon Oh, Je Hyuk Lee, Hyoung Moon Kim, Sosorburam Batsukh, Mi Jeong Sung, Tae Hwan Lim, Myoung Hoon Lee, Kuk Hui Son, Kyunghee Byun

**Affiliations:** 1 Functional Cellular Networks Laboratory, Lee Gil Ya Cancer and Diabetes Institute, Gachon University, Incheon 21999, Republic of Korea; 2 Doctorbom Clinic, Seoul 06614, Republic of Korea; 3 Department of Anatomy & Cell Biology, Gachon University College of Medicine, Incheon 21936, Republic of Korea; 4 SACCI Bio Co., Seoul 1007, Republic of Korea; 5 Department of Thoracic and Cardiovascular Surgery, Gachon University Gil Medical Center, Gachon University, Incheon 21565, Republic of Korea; 6 Department of Health Sciences and Technology, Gachon Advanced Institute for Health & Sciences and Technology (GAIHST), Gachon University, Incheon 21999, Republic of Korea

**Keywords:** poly-L-lactic acid, senescence, M2 polarization, collagen and elastin synthesis

## Abstract

Poly-L-lactic acid (PLLA) fillers correct cutaneous volume loss by stimulating fibroblasts to synthesize collagen and by augmenting the volume. PLLA triggers the macrophage-induced activation of fibroblasts that secrete transforming growth factor-β (TGF-β). However, whether M2 macrophage polarization is involved in PLLA-induced collagen synthesis via fibroblast activation in aged skin is not known. Therefore, we evaluated the effect of PLLA on dermal collagen synthesis via M2 polarization in an H_2_O_2_-induced cellular senescence model and aged animal skin. H_2_O_2_-treated macrophages had increased expression levels of the M1 marker CD80 and decreased expression levels of the M2 marker CD163, which were reversed by PLLA. The expression levels of interleukin (IL)-4 and IL-13, which mediate M2 polarization, were decreased in H_2_O_2_-treated macrophages and increased upon the PLLA treatment. CD163, IL-4, and IL-13 expression levels were decreased in aged skin, but increased after the PLLA treatment. The expression levels of TGF-β, pSMAD2/SMAD2, connective tissue growth factor (CTGF), alpha-smooth muscle actin (α-SMA), collagen type 1A1 (COL1A1), and COL3A1 were also decreased in aged skin, but increased after the PLLA treatment. Moreover, PLLA upregulated phosphatidylinositol 3-kinase p85α (PI3-kinase p85α)/protein kinase B (AKT) signaling, leading to fibroblast proliferation. PLLA decreased the expression of matrix metalloproteinase (MMP) 2 and MMP3, which destroy collagen and elastin fibers in aged skin. The amount of collagen and elastin fibers in aged skin increased following the PLLA treatment. In conclusion, PLLA causes M2 polarization by increasing IL-4 and IL-13 levels and upregulating TGF-β expression and collagen synthesis in aged skin.

## 1. Introduction

Aging induces various changes in the skin, including wrinkles, atrophy, elastosis, and the loss of subcutaneous volume [1,2]. Dermal fillers can simply and easily correct volume loss [3]. Therefore, the interest in using dermal fillers to rejuvenate skin without surgery has rapidly increased [4,5,6]. Filler materials are naturally sourced from animals or synthetic materials. Frequently used synthetic filler materials include calcium hydroxyapatite, polymethyl methacrylate, poly-L-lactic acid (PLLA), and silicone [7]. The biocompatibility of PLLA, a biodegradable polymer, has been demonstrated in medicine for a long time as a component of various surgical materials, including sutures, nails, pins, screws, and surgical plates [8,9].

PLLA filler injected into the skin with a carrier solution immediately augments the volume at the injection site, which disappears rapidly as the carrier solution is absorbed by the tissue [10]. The remaining PLLA particles are degraded into lactic acid, which enhances collagen synthesis by fibroblasts and gradually increases the dermal thickness [11]. Immune cells recognize PLLA as a foreign body, and subclinical inflammatory, foreign body reactions can cause PLLA-induced collagen synthesis [12,13]. During foreign body reactions, the adsorption of plasma proteins into foreign bodies in the blood initiates monocyte recruitment, and monocytes differentiate into macrophages, which subsequently increase the level of macrophage adhesion [14]. These immune cells then form giant cells, induce fibroblast recruitment, and form fibrotic tissue [14]. Macrophages play important roles during PLLA-induced collagen synthesis by secreting various cytokines, such as interleukin (IL)-1, IL-6, IL-8, transforming growth factor-beta (TGF-β), and tumor necrosis factor-alpha (TNF-α). These cytokines are involved in the degradation of biomaterials and tissue remodeling [15]. Tissues around the PLLA injection site accumulate CD68+ macrophages and CD90+ fibroblasts. They also increase TGF-β1 and the tissue inhibitor of metalloproteinase 1 (TIMP1) levels, which promote the deposition of collagen I and III [16]. 

Macrophages are classified into two subtypes: M1 and M2 [17,18]. M1 macrophages defend against bacteria or viruses and are activated by various proinflammatory stimuli, including interferon-gamma, granulocyte-macrophage colony-stimulating factor, and lipopolysaccharides. They secrete various inflammatory cytokines, such as IL-1, IL-6, IL-12, and IL-23 [18,19]. M2 macrophages are activated by IL-4 and IL-13 and are involved in fibrosis and tissue remodeling by secreting IL-10 and TGF-β [20,21]. We previously reported increased M1 marker expression and decreased M2 marker expression levels in aged animal skin compared to those of young animal skin [22]. Moreover, the expression levels of proinflammatory cytokines (e.g., TNF-α) were higher and those of anti-inflammatory cytokines (e.g., IL-10) were lower in aged skin compared to their levels in the skin of young animal skins [22]. 

Matrix metalloproteinases (MMPs) destroy collagen and elastin fibers in the skin, causing wrinkles [23,24]. The expression levels of MMP2, MMP3, and MMP9 are higher in aged animal skin than they are in young animal skin [22]. The number and function of dermal fibroblasts also decrease during aging [25,26]. Aging fibroblasts have decreased proliferative ability and secrete more extracellular matrix (ECM)-degrading proteins such as MMPs and proinflammatory cytokines [26]. Moreover, senescent fibroblasts produce less collagen and elastin fibers [27].

Previous studies showed the effects of PLLA fillers on collagen synthesis, primarily in young animals [28,29,30]. However, the reports described above suggest that the function of macrophages and fibroblasts in subclinical foreign body reactions to PLLA fillers could differ when PLLA is injected into aged skin. Therefore, we evaluated the effect of PLLA fillers on collagen synthesis in aged mouse skin. We hypothesized that PLLA induces M2 polarization and that M2 macrophages have increased IL-10 and TGF-β levels, resulting in collagen synthesis and decreased levels of MMPs in the aged skin.

## 2. Materials and Methods

### 2.1. PLLA Preparation

#### 2.1.1. PLLA Synthesis 

L-lactide (18 g, 0.12 mol) (Corbion, Amsterdam, The Netherlands), 0.09 mL stannous octoate (Sigma-Aldrich, St. Louis, MO, USA), and 0.54 g (0.003 mol) of the initiator 1-dodecanol (Sigma-Aldrich) were placed in a 1 L double-jacketed reactor at room temperature. After oxygen and moisture removal, nitrogen was injected into the reactor for 5 min. The mixture was gradually heated to 120 °C for 1 h, and then stirred at 120 °C for 4 h. The synthesized PLLA was collected via vacuum filtration, and the unreacted substances were removed using ethanol. PLLA was dissolved at room temperature in 220 mL dichloromethane (99.5%; Samchun Chemical Co., Ltd., Seoul, Republic of Korea). The PLLA solution was diluted via the slow, dropwise addition of 320 mL of 1% polyvinyl alcohol (87–90%, mol wt 30,000–70,000; Sigma-Aldrich) using a glass pipette while stirring it at 2000 rpm with a shear mixer. The solution was filtered under a vacuum for 3 h at room temperature and centrifuged at 3000 rpm for 10 min at room temperature to separate PLLA. After removing the supernatant, polyvinyl alcohol that remained in PLLA was redispersed in 50 mL distilled water and centrifuged at 3000 rpm for 10 min at room temperature. Finally, PLLA polymer was obtained by drying it in an oven at 45 °C for 24 h [31,32,33,34].

#### 2.1.2. Scanning Electron Microscopy (SEM) Imaging for Surface Observations of PLLA 

PLLA was biodegraded in vitro for 1, 2, 3, and 4 weeks at 50 °C. Because SEM imaging is difficult in a solution state, PLLA powder and supernatant were separated after biodegradation. PLLA powder was dried at 45 °C for 24 h. For SEM, PLLA powder (25 mg) was mounted on SEM stubs with conductive copper tape, and then coated with platinum at 30 mW for 180 s under an argon atmosphere. The surface was observed at an AC voltage of 15 kV at room temperature using a scanning electron microscope (Carl Zeiss, Jena, Germany).

### 2.2. Experimental Models

#### 2.2.1. PLLA Treatment in H_2_O_2_-Induced Senescent Macrophages

Murine macrophages (RAW 264.7 cells, Korea Cell Line Bank, Seoul, Republic of Korea) were cultured and maintained in Dulbecco’s Modified Eagle Medium (HyClone-Cytiva, Marlborough, MA, USA) containing 10% fetal bovine serum (FBS; Gibco-Thermo Fisher Scientific, Rockford, IL, USA) and 1% penicillin/streptomycin (P/S; Welgene, Gyeongsan, Republic of Korea) in a 37 °C incubator with 5% CO_2_. Senescence was induced by treating the macrophages with 100 μM H_2_O_2_ for 2 h. Cells were washed with Dulbecco’s phosphate-buffered saline (DPBS), and then incubated in growth medium for 72 h. Senescent and non-senescent cells were treated with phosphate-buffered saline (PBS) or 200 μg/mL PLLA (SACCI Bio Co., Seoul, Republic of Korea) for 48 h, respectively. After treatment, cell lysates were collected using a cell scraper, and supernatant (conditioned medium; CM) was centrifuged to remove floating cells (Figure S1) and concentrated using an Amicon tube (Merck Millipore, Burlington, MA, USA) at 4000× *g* for 40 min at room temperature. The macrophages used in this study were passaged 12–15 times, and the in vitro model was generated when cells reached approximately 80% confluency.

#### 2.2.2. PLLA Treatment of H_2_O_2_-Induced Senescent Fibroblasts

Human fibroblasts (CCD-986sk; American Type Culture Collection, Manassas, VA, USA) were maintained in Iscove’s Modified Dulbecco’s Medium (Welgene), 10% FBS (Gibco-Thermo Fisher Scientific), and 1% P/S (Welgene) in a 37 °C incubator with 5% CO_2_. Senescence was induced by treating fibroblasts with 350 μM H_2_O_2_ for 1.5 h. Cells were washed with DPBS, and then incubated in fresh growth medium for 72 h to induce senescence [35]. Senescent and non-senescent fibroblasts were treated with PBS or 200 μg/mL PLLA for 48 h (Figure S2). After treatment, cell lysates were collected using a cell scraper for protein analysis. The fibroblasts used in this study were passaged 7–9 times, and the in vitro model was generated when the cells reached approximately 70% confluency.

#### 2.2.3. Treatment of Senescent Fibroblasts with CM from PLLA-Treated Senescent Macrophages

Fibroblast senescence was induced with H_2_O_2_, as described in Section 2.2.2. Senescent and non-senescent fibroblasts were treated with CM collected in Section 2.2.1. for 48 h (Figure S3). Then, cell lysates were collected using a cell scraper. The fibroblasts used in this study were passaged 7–9 times, and the in vitro model was generated when the cells reached approximately 70% confluency.

#### 2.2.4. PLLA Treatment in Young and Aged Mice 

This study was approved by the ethical board of the Center of Animal Care and Use and was conducted in accordance with the Institutional Animal Care and Use Committee at Gachon University (approval number LCDI-2021-0172). Six-week-old male and female C57BL/6 mice were purchased from Orient Bio (Seongnam, Republic of Korea). After a week of adaptation, the mice were mated, and the resulting pups were raised for 2–14 months under a controlled temperature (22 ± 5 °C), relative humidity (50 ± 10 %), and 12 h light-dark cycle. They had free access to a standard laboratory diet and water.

Mice were assigned to young and aged groups so that skin samples could be collected at 13 weeks and 14 months, respectively. Within each age group, mice were randomly divided into treatment groups (*n* = 3/treatment group/time point). Saline or 10 mg/mL PLLA (100 μL) was injected into the dermis at five different areas of the mouse’s backs (5 cm × 5 cm), and skin tissue was collected under isoflurane anesthesia (HANA Pharm Co., Ltd., Seoul, Republic of Korea) after 1, 3, and 28 days once the hair was shaved from the injection sites (Figure S4).

### 2.3. Senescence-Associated β-Galactosidase (SA-β-gal) Activity

SA-β-gal-positive cells were detected using an SA-β-gal staining kit (Cell Signaling Technology, Danvers, MA, USA). Cells were seeded in 6-well plates. As described in Section 2.2.1, Section 2.2.2 and Section 2.2.3, three types of in vitro models were established. After washing with PBS, cells were fixed in fixation solution for 15 min at room temperature. After washing with PBS, cells were stained with β-gal staining solution at 37 °C for 24 h. For statistical analysis, 2000 cells were counted for each cell dispersion from all experimental groups in triplicate. 

### 2.4. Western Blotting

Cells and skin tissues were lysed using radioimmunoprecipitation assay buffer containing protease and phosphatase inhibitors (EzRIPA lysis kit; TaKaRa, Tokyo, Japan). After sonication, samples were centrifuged at 14,000× *g* for 15 min at 4 °C. Protein concentration was determined using the bicinchoninic acid assay (Thermo Fisher Scientific). Equal amounts of protein were separated using 8–12% sodium dodecyl sulfate-polyacrylamide gel electrophoresis and transferred to polyvinylidene fluoride (PVDF) membranes previously activated with methanol. PVDF membranes were blocked with 5% skim milk at room temperature to prevent the binding of non-specific proteins. Membranes were then incubated with appropriately diluted primary antibodies (Table S1) at 4 °C overnight. Membranes were rinsed with tris-buffered saline containing 0.1% Tween 20 and incubated with secondary antibodies at room temperature for 1 h. Protein bands were visualized using an enhanced chemiluminescence solution (Cytiva) using LAS-4000 (Bio-Rad, Hercules, CA, USA). Individual protein expression values were quantified using Image J software (National Institutes of Health, NIH, Maryland, MD, USA) and normalized to beta-actin (Cell Signaling Technology) to control the differences in protein loading. Values for a single blot are expressed relative to the mean of the first group.

### 2.5. Enzyme-Linked Immunosorbent Assay (ELISA)

A 96-well plate was coated with a coating solution containing 0.6% sodium bicarbonate (Sigma-Aldrich) and 0.3% sodium carbonate (Sigma-Aldrich) in distilled water at 4 °C overnight. The plate was blocked for 4 h at room temperature using 5% skim milk. After washing with PBS containing 0.1% Tween 20 (TPBS), protein samples isolated using the EzRIPA lysis kit (TaKaRa) were added to the plate, which was incubated at 4 °C for 24 h. After washing with TPBS, the plate was incubated overnight with primary antibodies at 4 °C (Table S1). After the plate was washed, the plate was incubated with horseradish peroxidase-conjugated secondary antibodies at room temperature for 2 h. After washing, the samples were incubated with 3,3′,5,5′-tetramethylbenzidine (Sigma-Aldrich) for 15 min for color development. The reactions were stopped with an equal volume of 2 M H_2_SO_4_, and absorbance was measured at 450 nm using an ELISA plate reader (Multiskan SkyHigh Photometer; Thermo Fisher Scientific).

### 2.6. Immunocytochemistry

Cells were seeded in 8-well Lab-Tek II chamber slides (Nunc, St. Louis, MO, USA). Non-senescent or senescence-induced cells were treated with PLLA for 48 h. Cells were washed with PBS and fixed using cold 4% paraformaldehyde (Sigma-Aldrich) at room temperature for 15 min. The slides were incubated with normal serum (Vector laboratories, Newark, CA, USA) for 1 h to block non-specific binding, and then incubated with primary antibodies (Table S1) at 4 °C for 24 h. After washing with PBS, the slides were incubated with secondary antibodies (Invitrogen, Waltham, MA, USA) at room temperature for 1 h in the dark. After washing, nuclei were counterstained with 4′,6-diamidino-2-phenylindole (DAPI; Sigma-Aldrich). Coverslips were mounted using Vectashield mounting solution (Vector laboratories), and fluorescent images were collected using a confocal microscope (Carl Zeiss 710) at the Core Facility for cell-to-in vivo imaging. Confocal images were randomly captured for image analysis, and fluorescence intensity was analyzed using ZEN 2009 software (Carl Zeiss).

### 2.7. Staining with 3,3-Diaminobenzidine (DAB)

Paraformaldehyde-fixed skin tissues prepared using a tissue processor (Sakura Seiki Co., Ltd., Tokyo, Japan) and an embedding device (Sakura Seiki Co., Ltd.) were sectioned using a microtome (Thermo Fisher Scientific) at 5 μm. Antigen retrieval was performed using sodium citrate buffer (pH 6). After washing with PBS, the sections were blocked with normal serum (Vector Laboratories) for 1 h at room temperature to prevent non-specific binding. After blocking, sections were incubated with primary antibodies (Table S1) overnight at 4 °C, and then with the corresponding biotinylated-conjugated secondary antibodies (Vector Laboratories) at room temperature for 1 h. Nuclei were counterstained with hematoxylin (KP&T, Cheong Ju, Republic of Korea), and coverslips were mounted with DPX mountant (Sigma-Aldrich).

The slides were visualized using an optical microscope (Olympus, Tokyo, Japan) and scanned and captured with a slide scanner (Motic, Vancouver, Canada). DAB was analyzed with a threshold for brown using Image J software (NIH). Five regions of the same size were randomly selected and analyzed for each tissue image.

### 2.8. Proliferation Assay

To analyze the effects of PLLA on the proliferative potential of fibroblasts, 5000 fibroblasts were seeded in each well of a 96-well culture plate (SPL Life Sciences, Pocheon, Republic of Korea), and the in vitro model was generated as described in Section 2.2.2 and Section 2.2.3. Cell counting kit (CCK; TransGen Biotech Co., Ltd., Beijing, China) reagent was diluted with serum-free medium (1:9, *v*/*v*). The cells were incubated with diluted reagent for 4 h at 37 °C in a 5% CO_2_ incubator. Optical density was measured at 450 nm using a plate reader (Multiskan SkyHigh Photometer; Thermo Fisher Scientific).

### 2.9. Immunofluorescence

After antigen retrieval, skin tissues sections (5-μm-thick slices) were blocked with normal serum (Vector Laboratories) at room temperature for 1 h, and then incubated with primary antibodies (Table S1). After incubation, the sections were rinsed with PBS and incubated with fluorescence-conjugated secondary antibodies (Invitrogen) for 1 h in the dark. Nuclei were counterstained with DAPI (Sigma-Aldrich) at room temperature for 30 s. After washing with PBS, coverslips were mounted using Vectashield mounting medium (Vector Laboratories), and slides were analyzed via confocal microscopy (Carl Zeiss 710, Carl Zeiss) at the Core Facility for cell-to-in vivo imaging. Confocal images were randomly captured for image analysis, and fluorescence intensity was analyzed using ZEN 2009 software (Carl Zeiss).

### 2.10. Histological Analysis

#### 2.10.1. Masson’s Trichrome Staining

After deparaffinization, skin tissues (5-μm-thick slices) were incubated in Bouin solution (Scytek Laboratories, West Logan, UT, USA) for 1 h at 60 °C, and then rinsed with distilled water. The sections were then stained with Weigert’s solution of iron hematoxylin (KP&T) for 5 min, Biebrich scarlet acid fuchsin solution (Scytek Laboratories) for 5 min, phosphomolybdic-phosphotungstic acid solution (Scytek Laboratories) for 12 min, and aniline blue solution (Scytek Laboratories) for 3 min. The stained slides were mounted using DPX mountant and observed using an optical microscope (Olympus) equipped with a slide scanner (Motic). Collagen fibers are shown blue. Nuclei are shown blue-black, and the cytoplasm, keratin, and muscle fibers are shown in pink and red colors. The collagen fiber density in images was analyzed using Image J software (NIH).

#### 2.10.2. Herovici’s Staining

Mature collagen and newly generated collagen were identified in the skin tissues using Herovici’s stain kit (Scytek Laboratories). Deparaffinized slides were incubated for 8 min in Weigert’s iron hematoxylin to stain the nuclei, and then washed with tap water, followed by distilled water. Slides were treated with Herovici’s solution for 2 min, mounted using DPX mountant, and visualized using an Olympus microscope equipped with a slide scanner (Motic). The blue stained fibers in all images representing newly synthesized collagen fibers and the yellow-light red-stained fibers representing mature collagen were quantified in all images using Image J software (NIH).

#### 2.10.3. Verhoeff’s Staining

Deparaffinized skin tissues were treated with elastic stain solution (Scytek Laboratories) for 15 min at room temperature, and then rinsed with tap water. Slides were incubated with 20 drops of 2% ferric chloride differentiation solution (Scytek Laboratories), washed with distilled water, and mounted using DPX mountant. Slides were visualized using an optical microscope equipped with a slide scanner (Motic). The density of the elastin fibers blue-black in the papillary dermis was analyzed in the captured images using Image J software (NIH). 

### 2.11. Statistical Analysis

The Kruskal–Wallis test, followed by the Mann–Whitney U post hoc test, was performed to compare the groups. The results of this study were validated by the unpaired *t*-test using SPSS software version 22 (IBM Corporation, Armonk, NY, USA). All results are presented as the mean ± standard deviation, and all experiments were repeated in triplicate. Statistical significance is described in the respective figure legends.

## 3. Results

### 3.1. Morphology and Degradation Pattern of PLLA

**Figure 1:**
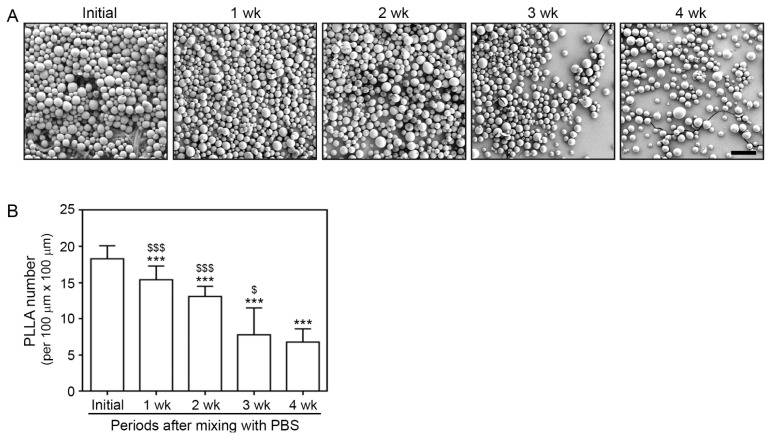
The number of PLLA decreased over time, whereas its spherical shape was maintained. (**A**) PLLA morphology was confirmed via SEM. PLLA particles maintained their round shape for four weeks and gradually decreased in number (scale bar = 100 μm). (**B**) Graph quantifying the number of PLLA per 100 μm × 100 μm area counted from randomly selected areas. Data are presented as the mean ± SD. A different alphabet indicates differences in statistical significance between groups. ***, *p* < 0.001, first bar vs. the others bar; $ and $$$, *p* < 0.05 and *p* < 0.001, fifth bar vs. second, third, and fourth bar. PLLA, poly-L-lactic acid; SD, standard deviation; SEM, scanning electron microscopy; wk, weeks.

The morphology of PLLA particles was visualized via SEM every week for four weeks. The initial appearance of PLLA particles was observed immediately after mixing them with PBS (Figure 1A). The PLLA particles were round with a smooth surface. The number of PLLA particles decreased over time, whereas their round surface morphology was maintained (Figure 1A). The degradation rate decreased from 3 to 4 weeks after mixing (Figure 1B). 

### 3.2. PLLA Increased IL-4 and IL-13 Expression and M2 Polrization in H_2_O_2_-Treated Macrophages and Aged Skin

H_2_O_2_-induced cellular senescence is most frequently used in vitro aging models [36]. Therefore, we used it in this model to evaluate the effect of PLLA on senescent cells. After the induction of senescence, we treated the induced and uninduced cells with PLLA or PBS to compare the responses of senescent and non-senescent macrophages to PLLA (Figure S1).

SA-β-gal, p21, and p16 are well-known markers of cellular senescence [37]. The levels of SA-β-gal activity and p16 expression were not significantly different in PBS-treated macrophages after PLLA administration. In contrast, H_2_O_2_-treated macrophages had higher levels of SA-β-gal activity and p16 expression than the PBS-treated macrophages did, which PLLA administration decreased in the H_2_O_2_-treated macrophages. H_2_O_2_-treated macrophages had an increased p21 expression level compared to that of the PBS-treated macrophages. PLLA reduced the level of p21 expression in both PBS-treated and H_2_O_2_-treated macrophages (Figure S5).

**Figure 2:**
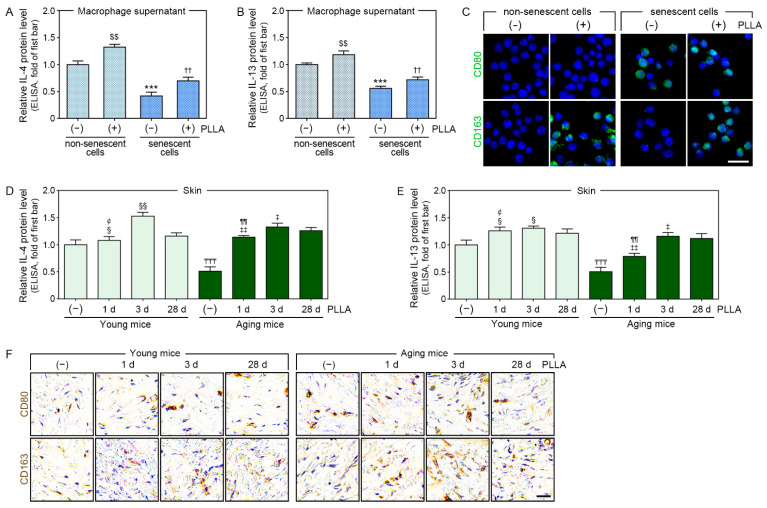
PLLA increased IL-4 and IL-13 secretion, leading to M2 polarization. H_2_O_2_-induced senescent or non-senescent macrophages were treated with PBS (first or third bar) or PLLA (second or fourth bar). (**A**,**B**) IL-4 and IL-13 levels in supernatant (CM) from non-senescent and H_2_O_2_-induced senescent macrophages were measured via ELISA. (**C**) CD80 (M1 marker) and CD163 (M2 marker) levels in H_2_O_2_-induced senescent and non-senescent macrophages were analyzed by immunocytochemistry (green: positive signals, blue: nuclei; scale bar = 20 μm). Young or aging mice were injected saline or PLLA and skin samples were collected after 1 (2nd or 6th bar), 3 (3rd or 7th bar), and 28 days (1st, 4th, 5th, or 8th bar). (**D**,**E**) IL-4 and IL-13 levels in young and aged skin were measured via ELISA. (**F**) CD80 and CD163 expression levels in young and aged skin were analyzed via DAB staining (scale bar = 50 μm). Data are presented as the mean ± SD (*n* = 3/group). $$, *p* < 0.01, first bar vs. second bar in (**A**,**B**); ***, *p* < 0.001, first bar vs. third bar in (**A**,**B**); ††, *p* < 0.01, third bar vs. fourth bar in (**A**,**B**); ₸₸₸, *p* < 0.001, first bar vs. fifth bar in (**D**,**E**); ¢, *p* < 0.05, third bar vs. second bar in (**D**,**E**); § and §§, *p* < 0.05 and *p* < 0.01, fourth bar vs. second or third bar in (**D**,**E**); ¶¶, *p* < 0.01, seventh bar vs. sixth bar in (**D**,**E**); ‡ and ‡‡, *p* < 0.05 and *p* < 0.01, eighth bar vs. sixth or seventh bar in (**D**,**E**). CD80, cluster of differentiation 80; CD163, cluster of differentiation 163; CM, conditioned medium; d, days; DAB, 3,3′-diaminobenzidine; ELISA, enzyme-linked immunosorbent assay; H_2_O_2_, hydrogen peroxide; IL-4, interleukin-4; IL-13, interleukin-13; PBS, phosphate-buffered saline; PLLA, poly-L-lactic acid; SD, standard deviation.

We then used this senescence model to evaluate whether PLLA increased the IL-4 and IL-13 levels and indued M2 polarization. IL-4 and IL-13 levels were evaluated in the CM from macrophage cultures via ELISA. The H_2_O_2_ treatment decreased the levels of these cytokines; however, the PLLA treatment abrogated this effect. Moreover, the IL-4 and IL-13 levels increased in PBS-treated macrophages following a treatment with PLLA (Figure 2A,B).

The expression levels of CD80 and CD163 (M1 and M2 markers, respectively [38]) were also evaluated in the macrophages. The H_2_O_2_ treatment increased the CD80 expression level, which was significantly decreased by PLLA. However, PLLA did not cause changes in CD80 expression in PBS-treated macrophages (upper rows of Figure 2C and Figure S6A). PLLA also increased the CD163 expression levels that had been reduced by the H_2_O_2_ treatment. In addition, the CD163 expression levels increased in PBS-treated macrophages following the PLLA treatment (lower rows of Figure 2C and Figure S6B).

We then evaluated the expression levels of IL-4, IL-13, and M1 and M2 markers in saline-injected animal skin and compared them between young and aged animals at 28 days after the injection. In addition, the expressions levels of these molecules were compared with those of PLLA-treated young and aged animals at 1, 3, and 28 days after the injection. We found that the IL-4 and IL-13 expression levels in saline-injected aged skin were lower than those in saline-injected young skin. The expression of these cytokines peaked three days after the PLLA injection in both young and aged animals (Figure 2D,E).

The CD80 expression level was significantly higher in saline-injected aged animals than it was in saline-injected young animals. In young animals, the CD80 expression level peaked three days after the PLLA injection and decreased at day 28. The level of CD80 expression also peaked three days after the PLLA injection for the aged animals. The extent of the change in CD80 expression over time was less pronounced in the aged animals than it was in the young animals (upper rows of Figure 2F and Figure S6C). 

The CD163 expression level in saline-injected aged skin was lower than it was in saline-injected young skin. Its expression peaked three days after PLLA injection in young animals and decreased at day 28. The CD163 expression level 28 days after the PLLA injection was higher than its expression level after the saline injection. Its expression also peaked three days after the PLLA injection in aged animals. The CD163 expression level was higher in the aged animals than it was in the young animals at identical time points (lower rows of Figure 2F and Figure S6D). 

### 3.3. PLLA Upregulated Expression of TGF-β, pSMAD2/SMAD2, and Connective Tissue Growth Factor (CTGF) in H_2_O_2_-Treated Fibroblasts 

We evaluated whether PLLA could improve collagen synthesis by directly stimulating fibroblasts without macrophages involvements. To this end, we induced fibroblast senescence with H_2_O_2_, and then treated the senescent cells with PBS or PLLA (Figure S2). SA-β-gal activity and p21 expression were not significantly changed in PBS-treated fibroblasts following PLLA administration. H_2_O_2_-treated fibroblasts had higher SA-β-gal activity and p21 expression levels than the PBS-treated fibroblasts did. PLLA caused a decrease in these markers in H_2_O_2_-treated fibroblasts (Figure S7A–C). The PLLA treatment decreased the p16 expression level in the PBS-treated fibroblasts. H_2_O_2_-treated fibroblasts had higher p16 expression levels than PBS-treated fibroblasts did. PLLA decreased the level of p16 expression in H_2_O_2_-treated fibroblasts (Figure S7B,D).

TGF-β, pSMAD2/SMAD2, CTGF, alpha-smooth muscle actin (α-SMA), collagen type 1A1 (COL1A1), and COL3A1 levels were also evaluated in fibroblast lysates. 

**Figure 3:**
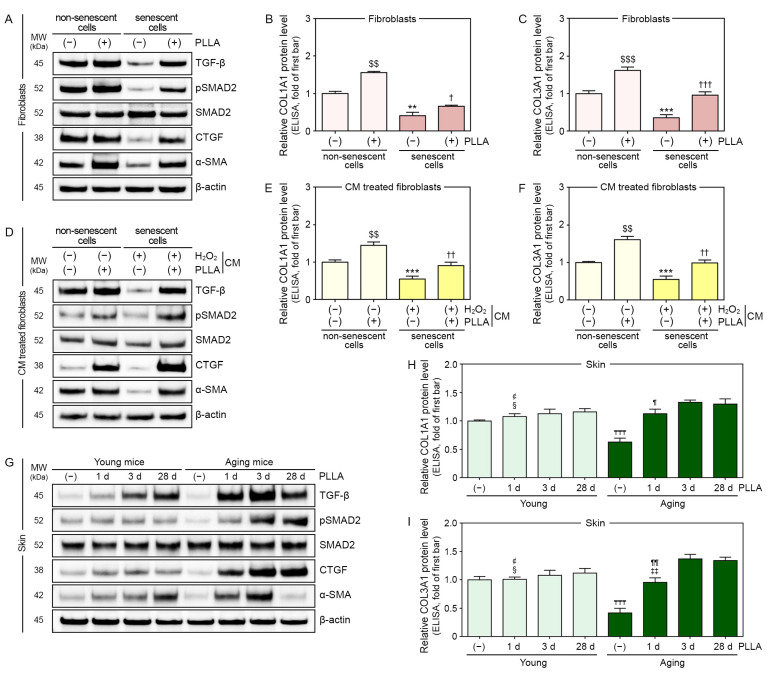
PLLA induced collagen synthesis in fibroblasts and aged animal skin. H_2_O_2_-induced senescent or non-senescent fibroblasts were treated with PBS (first or third bar) or PLLA (second or fourth bar). (**A**) TGF-β, SMAD2, pSMAD2, CTGF, and α-SMA levels in H_2_O_2_-induced senescent and non-senescent fibroblasts were analyzed via Western blotting. (**B**,**C**) COL1A1 and COL3A1 levels in H_2_O_2_-induced senescent and non-senescent fibroblasts were measured via ELISA. Senescent or non-senescent fibroblasts were treated with CM from macrophages treated with PBS (first bar), PBS/PLLA (second bar), H_2_O_2_/PBS (third bar), or H_2_O_2_/PLLA (fourth bar). (**D**) TGF-β, SMAD2, pSMAD2, CTGF, and α-SMA levels in H_2_O_2_-induced senescent and non-senescent fibroblasts were analyzed via Western blotting. (**E**,**F**) COL1A1 and COL3A1 levels in H_2_O_2_-induced senescent and non-senescent fibroblasts were measured via ELISA. Young or aging mice were injected saline or PLLA and skin samples were collected after 1 (2nd or 6th bar), 3 (3rd or 7th bar), and 28 days (1st, 4th, 5th, or 8th bar). (**G**) TGF-β, SMAD2, pSMAD2, CTGF, and α-SMA levels in young and aged skin were analyzed via Western blotting. (**H**,**I**) COL1A1 and COL3A1 levels in young and aged skin were measured via ELISA. Data are presented as the mean ± SD (*n* = 3/group). $$ and $$$, *p* < 0.01 and *p* < 0.001, first bar vs. second bar in (**B**,**C**,**E**,**F**); ** and ***, *p* < 0.01 and *p* < 0.001, first bar vs. third bar in (**B**,**C**,**E**,**F**); †, †† and †††, *p* < 0.05, *p* < 0.01 and *p* < 0.001, third bar vs. fourth bar in (**B**,**C**,**E**,**F**); ₸₸₸, *p* < 0.001, first bar vs. fifth bar in (**H**,**I**); ¢, *p* < 0.05 and *p* < 0.01, third bar vs. second bar in (**H**,**I**); §, *p* < 0.05, fourth bar vs. second or third bar in (**H**,**I**); ¶ and ¶¶, *p* < 0.05 and *p* < 0.01, seventh bar vs. sixth bar in (**H**,**I**). α-SMA, alpha-smooth muscle actin; β-actin, beta-actin; CM, conditioned medium; COLA1A, collagen type 1A1; COLA3A1, collagen type 3A1; CTGF, connective tissue growth factor; d, days; ELISA, enzyme-linked immunosorbent assay; H_2_O_2_, hydrogen peroxide; kDa, kilodalton; MW, molecular weight; PBS, phosphate-buffered saline; PLLA, poly-L-lactic acid; pSMAD2, phosphorylated SMAD2; SD, standard deviation; TGF-β, transforming growth factor-beta.

TGF-β, pSMAD2/SMAD2, CTGF, and α-SMA levels were lower in H_2_O_2_-treated fibroblasts than they were in PBS-treated fibroblasts. TGF-β and CTGF were unchanged by the PLLA treatment in PBS-treated fibroblasts, but the levels were increased in H_2_O_2_-treated fibroblasts. In contrast, pSMAD2/SMAD2 and α-SMA levels were increased in both of PBS- and H_2_O_2_-treated fibroblasts by PLLA (Figure 3A and Figure S8).

The levels of COL1A1 and COL3A1 were lower in H_2_O_2_-treated fibroblasts than they were in PBS-treated fibroblasts, and these levels increased following the PLLA treatment in both fibroblast groups (Figure 3B,C).

### 3.4. PLLA Upregulated the Expression of TGF-β, pSMAD2/SMAD2, and CTGF in Fibroblasts Treated with CM from H_2_O_2_-Treated Macrophages and Aged Skin

Next, we evaluated whether PLLA improved the collagen synthesis in fibroblasts via macrophage modulation. Macrophages were treated with PBS, and then with PBS or PLLA. CM from PBS/PBS- or PBS/PLLA-treated macrophages was administered to PBS-treated fibroblasts. CM from H_2_O_2_/PBS- or H_2_O_2_/PLLA-treated macrophages was administered to H_2_O_2_-treated fibroblasts (Figure S3).

SA-β-gal activity and the expression of p21 and p16 in PBS-treated fibroblasts were unchanged by the treatment with CM from PBS/PLLA-treated macrophages compared to the levels of these markers in PBS-treated fibroblasts incubated with CM from PBS/PBS-treated macrophages. However, these parameters were decreased in H_2_O_2_-treated fibroblasts incubated with CM from H_2_O_2_/PLLA-treated macrophages compared to those treated with CM from H_2_O_2_/PBS-treated macrophages (Figure S9). 

TGF-β, pSMAD2/SMAD2, CTGF, and α-SMA expression levels in PBS- and H_2_O_2_-treated fibroblasts were increased following the treatment with CM from PBS/PLLA- or H_2_O_2_/PLLA-treated macrophages (Figure 3D and Figure S10).

COL1A1 and COL3A1 levels were also increased in PBS- and H_2_O_2_-treated fibroblasts by the treatment with CM from PBS/PLLA- or H_2_O_2_/PLLA-treated macrophages (Figure 3E,F).

The TGF-β expression level in saline-injected aged skin was significantly lower than it was in saline-injected young skin, and it was increased in both young and aged skin following PLLA injection. In PLLA-injected young skin, the TGF-β expression level peaked 28 days after PLLA injection; however, it peaked three days after PLLA injection in aged skin (Figure 3G and Figure S11A). 

The pSMAD2/SMAD2 ratio and CTGF for saline-injected aged skin was significantly lower than it was for saline-injected young skin. This ratio in PLLA-injected young skin did not change over time; however, it peaked three days after PLLA injection in aged skin (Figure 3G and Figure S11B).

CTGF and α-SMA levels were significantly lower in saline-injected aged skin than they were in saline-injected young skin. In PLLA-injected young skin, CTGF expression did not change over time; however, it peaked three days after PLLA injection into aged skin (Figure 3G and Figure S11C). In PLLA-injected young skin, α-SMA expression peaked 28 days after PLLA injection. However, α-SMA expression peaked three days after PLLA injection in aged skin (Figure 3G and Figure S11D).

COL1A1 and COL3A1 levels in saline-injected aged skin were lower than they were in saline-injected young skin. COL1A1 expression peaked 3 and 28 days after PLLA injection in young and aged skin, respectively (Figure 3H). COL3A1 expression levels on day 3 and day 28 post-PLLA injection were higher than they were on day 1 after the injection in both young and aged skin (Figure 3I). 

### 3.5. PLLA Upregulated Phosphatidylinositol 3-Kinase p85α (PI3-Kinase p85α)/Protein Kinase B (AKT) Signaling and Fibroblast Proliferation

**Figure 4:**
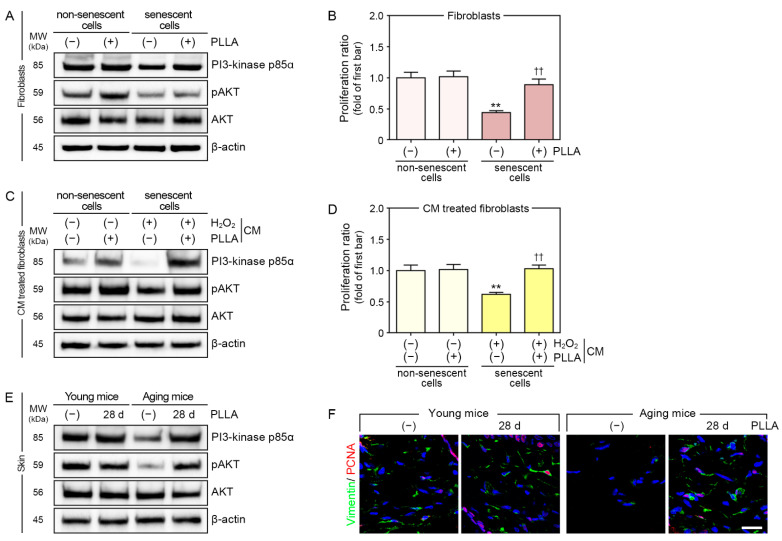
PLLA induced proliferation in fibroblasts and aged animal skin. H_2_O_2_-induced senescent or non-senescent fibroblasts were treated with PBS (first or third bar) or PLLA (second or fourth bar). (**A**) PI3-kinase p85α, pAKT/AKT levels in H_2_O_2_-induced senescent and non-senescent fibroblasts were analyzed via Western blotting. (**B**) The proliferation ratio for H_2_O_2_-induced senescent and non-senescent fibroblasts were measured using the proliferation assay. Senescent or non-senescent fibroblasts were treated with CM from macrophages treated with PBS (first bar), PBS/PLLA (second bar), H_2_O_2_/PBS (third bar), or H_2_O_2_/PLLA (fourth bar). (**C**) PI3-kinase p85α, pAKT/AKT levels in H_2_O_2_-induced senescent and non-senescent fibroblasts were analyzed via Western blotting. (**D**) The proliferation ratio for H_2_O_2_-induced senescent and non-senescent fibroblasts was measured using the proliferation assay. Young or aging mice were injected saline or PLLA and skin samples were collected after 1 and 28 days. (**E**) PI3-kinase p85α, pAKT/AKT levels in young and aged skin were analyzed via Western blotting. (**F**) The expression of vimentin (fibroblast marker, green) and PCNA (proliferation marker, red) in young and aged skin was analyzed using immunofluorescence (nuclei, blue) (scale bar = 50 μm). Data are presented as the mean ± SD (*n* = 3/group). **, *p* < 0.01, first bar vs. third bar in (**B**,**D**); ††, *p* < 0.01, third bar vs. fourth bar in (**B**,**D**). β-actin, beta-actin; CM, conditioned medium; H_2_O_2_, hydrogen peroxide; kDa, kilodalton; MW, molecular weight; pAKT, phosphorylated AKT; PBS, phosphate-buffered saline; PCNA, proliferating cell nuclear antigen; PLLA, poly-L-lactic acid; SD, standard deviation.

Fibroblast proliferation is induced by the upregulation of PI3-kinase p85α/AKT signaling [39]. Therefore, we evaluated PI3-kinase p85α and pAKT/AKT expression and the proliferation of PBS- or H_2_O_2_-treated fibroblasts after administering PBS or PLLA (Figure 4A,B).

The PI3-kinase p85α expression level was decreased in fibroblasts treated with H_2_O_2_. In PBS-treated fibroblasts, the PI3-kinase p85α expression level was unchanged after the PLLA treatment, whereas its expression increased in H_2_O_2_-treated fibroblasts after the PLLA treatment (Figure 4A and Figure S12A). 

The pAKT/AKT ratio decreased in fibroblasts treated with H_2_O_2_; however, this ratio increased after the PLLA treatment in both PBS- and H_2_O_2_-treated fibroblasts (Figure 4A and Figure S12B).

The level of fibroblast proliferation was also decreased by H_2_O_2_. The PLLA treatment did not change the proliferation of PBS-treated fibroblasts, but it increased H_2_O_2_-treated fibroblast proliferation (Figure 4B).

In PBS-treated fibroblasts, PI3-kinase p85α and pAKT/AKT levels were increased by incubation with CM from PBS/PLLA-treated macrophages. The levels of these proteins also increased when H_2_O_2_-treated fibroblasts were incubated with CM from H_2_O_2_/PLLA-treated macrophages (Figure 4C and Figure S12C,D). CM from PBS/PLLA-treated macrophages did not alter PBS-treated fibroblast proliferation; however, the proliferation of H_2_O_2_-treated fibroblasts increased after exposure to CM from H_2_O_2_/PLLA-treated macrophages (Figure 4D). 

The PI3-kinase p85α expression level decreased more in saline-injected aged skin compared to that in saline-injected young skin; however, it increased following the PLLA injection in both skin types (Figure 4E and Figure S12E). 

The pAKT/AKT ratio decreased more in saline-injected aged skin compared to that in saline injected young skin, and it did not change in young skin after the PLLA injection; however, it did increase in aged skin as a result of the PLLA injection (Figure 4E and Figure S12F).

Staining for proliferating cell nuclear antigen (PCNA) was also performed to evaluate fibroblast proliferation in the dermis. The number of fibroblasts stained with PCNA was lower in saline-injected aged skin than it was in saline-injected young skin. PLLA injection did not change the number of PCNA+ fibroblasts in young skin, but it increased their numbers in aged skin (Figure 4F and Figure S12G).

### 3.6. PLLA Increased the Expression of IL-10 and TIMP1 and Decreased the Expression of MMP2 and MMP3

M2 macrophages secrete IL-10, suppress inflammatory reactions, and promote tissue remodeling and repair [40]. TIMPs inhibit secreted MMPs, thereby decreasing the degradation of extracellular matrix (ECM) components such as collagen and elastin fibers [41]. 

**Figure 5:**
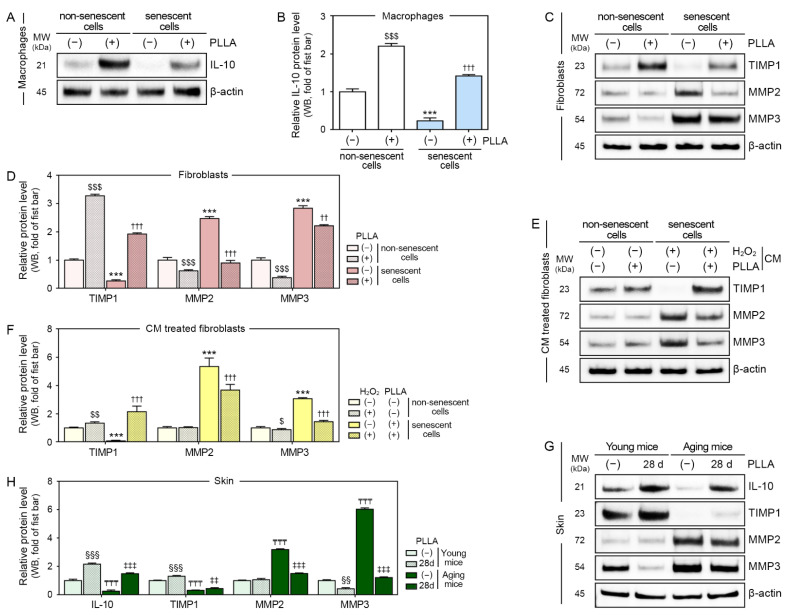
PLLA inhibited collagen degradation in senescent fibroblasts and aged animal skin. H_2_O_2_-induced senescent or non-senescent macrophages were treated with PBS (first or third bar) or PLLA (second or fourth bar). (**A**) IL-10 expression in H_2_O_2_-induced senescent and non-senescent macrophages were analyzed via Western blotting. (**B**) Graph quantifying the data in (**A**). H_2_O_2_-induced senescent or non-senescent fibroblasts were treated with PBS (first or third bar) or PLLA (second or fourth bar). (**C**) TIMP1, MMP2, and MMP3 levels in H_2_O_2_-induced senescent and non-senescent fibroblasts were analyzed via Western blotting. (**D**) Graph quantifying the data in (**C**). Senescent or non-senescent fibroblasts were treated with CM from macrophages treated with PBS (first bar), PBS/PLLA (second bar), H_2_O_2_/PBS (third bar), or H_2_O_2_/PLLA (fourth bar). (**E**) TIMP1, MMP2, and MMP3 levels in H_2_O_2_-induced senescent and non-senescent fibroblasts were analyzed via Western blotting. (**F**) Graph quantifying the data in (**E**). Young or aging mice were injected saline or PLLA and skin samples were collected after 1 (first or third bar) and 28 days (second or fourth bar). (**G**) IL-10, TIMP1, MMP2, and MMP3 levels in young and aged skin were analyzed via Western blotting. (**H**) Graph quantifying the data in (**G**). Data are presented as the mean ± SD (*n* = 3/group). $, $$ and $$$, *p* < 0.05, *p* < 0.01 and *p* < 0.001, first bar vs. second bar in (**B**,**D**,**F**); ***, *p* < 0.001, first bar vs. third bar in (**B**,**D**,**F**); †† and †††, *p* < 0.01 and *p* < 0.001, third bar vs. fourth bar in (**B**,**D**,**F**); §§ and §§§, *p* < 0.01 and *p* < 0.001, first bar vs. second bar in (**H**); ₸₸₸, *p* < 0.001, first bar vs. third bar in (**H**); ‡‡, ‡‡‡, *p* < 0.01, and *p* < 0.001 third bar vs. fourth bar in (**H**). β-actin, beta-actin; CM, conditioned medium; H_2_O_2_, hydrogen peroxide; IL-10, interleukin-10; kDa, kilodalton; MMP2, matrix metalloproteinase 2; MMP3, matrix metalloproteinase 3; MW, molecular weight; PBS, phosphate-buffered saline; PLLA, poly-L-lactic acid; SD, standard deviation; TIMP1, tissue inhibitor of metalloproteinase 1; WB, Western blotting.

In this study, the IL-10 levels in macrophages were decreased by the H_2_O_2_ treatment and increased by the PLLA treatment in both PBS- and H_2_O_2_-treated macrophages (Figure 5A,B). 

TIMP1 levels in the fibroblasts were decreased by H_2_O_2_ treatment, but they were increased in PBS- and H_2_O_2_-treated fibroblasts after the PLLA treatment. The expression of MMP2 and MMP3 produced the opposite pattern (Figure 5C,D).

In PBS- and H_2_O_2_-treated fibroblasts, the TIMP1 levels increased after the treatment with CM from PBS/PLLA- or H_2_O_2_/PLLA-treated macrophages, respectively. In PBS-treated fibroblasts, the MMP2 levels remained unchanged by the treatment with CM from PBS/PLLA-treated macrophages. In contrast, the MMP2 levels were decreased in H_2_O_2_-treated fibroblasts by CM from H_2_O_2_/PLLA-treated macrophages. The MMP3 levels were decreased in PBS-treated fibroblasts by CM from PBS/PLLA-treated macrophages. In H_2_O_2_-treated fibroblasts, the MMP3 levels were decreased by the CM treatment from H_2_O_2_/PLLA-treated macrophages (Figure 5E,F).

IL-10 and TIMP1 expression levels in saline-injected aged skin were lower than they were in saline-injected young skin, and PLLA increased the levels of these two proteins in both skin types (Figure 5G,H).

MMP2 and MMP3 levels were higher in saline-injected aged skin than they were in saline-injected young skin. The PLLA treatment did not alter MMP2 expression in young skin, but it decreased its expression in aged skin. The PLLA treatment decreased the MMP3 expression level in both young and aged skin (Figure 5G,H).

### 3.7. PLLA Increased Collagen and Elastin Fiber Content in Aged Skin

**Figure 6:**
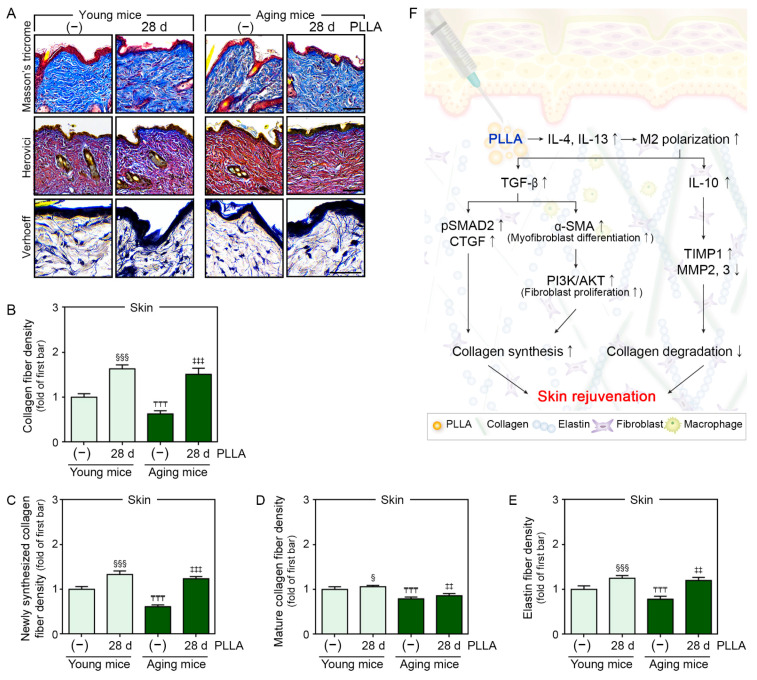
PLLA increased new collagen and elastin fibers. Young or aging mice were injected with saline or PLLA, and skin samples were collected after 1 (first or third bar) and 28 days (second or fourth bar). (**A**) Masson’s trichrome, Herovici’s, and Verhoeff’s staining of young and aged skin (scale bar = 50 µm). (**B**–**E**) Quantification of the data shown in (**A**). The densities of collagen fibers (**B**), newly synthesized collagen fibers (**C**), mature collagen fibers (**D**), and elastic fibers (**E**) decreased in aged skin compared to those in young skin and increased in both skin types following PLLA treatment. Data are presented as the mean ± SD (*n* = 3/group). § and §§§, *p* < 0.05 and *p* < 0.001, first bar vs. second bar; ₸₸₸, *p* < 0.001, first bar vs. third bar; ‡‡, ‡‡‡, *p* < 0.01, and *p* < 0.001 third bar vs. fourth bar. (**F**) Summary of the study. PLLA, poly-L-lactic acid; SD, standard deviation.

Collagen fibers in young and aged skin 28 days after saline or PLLA injection were identified via Masson’s trichrome staining. The collagen fiber density of saline-injected aged skin was significantly lower than that of saline-injected young skin. The PLLA treatment increased the collagen fiber density in both skin types (first lane of Figure 6A,B).

To determine if PLLA affected newly synthesized collagen or mature collagen, young and aged skin sections were stained using Herovici’s stain. Herovici’s collagen staining was performed to differentiate newly synthesized collagen (stained blue) from mature collagen (stained red) [42,43]. The newly synthesized and mature collagen levels in saline-injected aged skin were significantly lower than they were in saline-injected young skin. The PLLA treatment increased the amount of new and mature collagen in both skin types (second lane of Figure 6A,C,D).

The amount elastin fibers also increased through TGF-β/SMAD signaling in the skin [44]. Therefore, we evaluated elastin fibers, the main component of the skin’s ECM, using Verhoeff’s stain. The elastin fiber density was significantly lower in saline-injected aged skin than it was in saline-injected young skin. The PLLA treatment increased the elastin fiber content in both cell types (last lane of Figure 6A,E).

## 4. Discussion

During aging, the thickness of the dermis decreases, while the degree of fragmentation of collagen fibers in the dermis increases [45]. Moreover, the amount of collagen and elastin fibers gradually decreases due to cutaneous aging [46].

PLLA is a stimulatory filler that can stimulate collagen synthesis even after degradation, and it is, therefore, different from traditional fillers, which only augment volume [47]. Because cutaneous aging is accompanied by decreased collagen and elastin fiber contents, PLLA fillers might effectively correct the volume loss in aged skin by inducing collagen synthesis and augmenting the volume. Moreover, PLLA fillers promote collagen synthesis via a foreign body reaction mainly initiated by macrophages [48]. PLLA fillers cannot induce collagen synthesis when fibroblasts are cultured in the absence of macrophages, but PLLA increases the level of TGF-β expression and induces collagen synthesis in fibroblasts when they are cocultured with macrophages [49].

However, the effect of PLLA administration on macrophage polarization has not been evaluated. M2 macrophages stimulate the proliferation of dermal fibroblasts when these cell types are cocultured; however, M1 macrophages do not stimulate dermal fibroblast proliferation. Moreover, the synthesis of collagen I and III is induced by M2, but not by M1 macrophages [50]. Furthermore, M2 polarization improves the rates of epidermal recovery and tissue regeneration by inducing proper tissue remodeling [51]. The ratio of M1 to M2 macrophages increases in aged skin, and an increased proportion of M1 macrophages is associated with increased skin inflammation [52]. CM from M1 macrophages increases the number of SA-β-gal-positive dermal fibroblasts, suggesting that M1 macrophages promote the senescence of dermal fibroblasts, whereas CM from M2 macrophages inhibits senescence [52].

PLLA fillers might be involved in modulating M2 polarization. In addition, the induction of foreign body reactions by PLLA could be different in aged skin because aging affects both macrophages and fibroblasts. Therefore, we hypothesized that PLLA could induce polarization toward the M2 subtype, stimulating fibroblasts to synthesize collagen by increasing the level of TGF-β expression. We then evaluated whether PLLA could increase the amount of collagen and elastin fibers even in aged animal skin.

The PLLA particles used in the present study had a round shape and very smooth surface, which were maintained during the degradation process for four weeks. Immune cells generally recognize particles based on the surface properties of their constituent materials [53]. Particles with spiky surfaces induce the higher secretion of IL-1β and inflammasome formation than particles with smooth surfaces do [54]. Fiber-shaped materials increase the levels of IL-6 expression and M1 polarization more than spherical materials do [55]. We did not compare the expression of M1 markers based on the shape of PLLA particles in the present study. Therefore, we could not provide conclusive evidence on whether particle shape is associated with the modulation of M2 polarization. However, maintaining a round shape with a smooth surface during degradation might be advantageous for PLLA particles because round shapes might decrease the expression of M1 markers.

In the present study, PLLA promoted the expression of IL-4 and IL-13. IL-13 triggers immune cells, specifically macrophages, to secrete TGF-β1 and directly acts on myofibroblasts to synthesize the ECM [56]. TGF-β1 plays various roles in promoting ECM synthesis. TGF-β1 enhances the differentiation of fibroblasts to myofibroblasts that express α-SMA and promotes the synthesis of ECM components such as collagen and elastin [57]. Moreover, TGF-β1 promotes the proliferation of fibroblasts and differentiation into myofibroblasts via the PI3-kinase p85α/AKT pathway [58,59]. TGF-β1 upregulates SMAD2 and SMAD3, leading to the increased rate of synthesis of CTGF and ECM [57].

It has been reported that culturing fibroblasts with PLLA cannot stimulate collagen synthesis [50]. Thus, we evaluated whether PLLA could directly stimulate fibroblasts without the involvement of macrophages. Our results showed that PLLA increased the expression level of several factors that cause collagen synthesis, such as TGF-β, pSMAD2/SMAD2, and CTGF, in fibroblasts in the absence of macrophages-CM. However, these PLLA-induced changes were observed more prominently in senescent fibroblasts than they were in non-senescent fibroblasts. The expression levels of α-SMA, which induces the differentiation of fibroblasts to myofibroblasts, and pAKT/AKT were increased by PLLA in both senescent and non-senescent fibroblasts. The proliferation ratio and PI3-kinase p85α levels were only increased in senescent fibroblasts by PLLA. The previous study did not use a senescence model, and the type of PLLA in the previous report differed from the type of PLLA used in the present study. These differences might lead to different PLLA effects on signal transduction pathways related to collagen synthesis in fibroblasts when fibroblasts are exposed to PLLA without exposure to macrophages.

We also analyzed whether PLLA could initially modulate macrophages that, in turn, stimulate collagen synthesis in fibroblasts using CM from non-senescent and senescent macrophages. Our results suggested that PLLA modulated senescent macrophages, which caused senescent fibroblasts to increase their TGF-β, pSMAD2/SMAD2, and CTGF levels. Moreover, PLLA similarly modulated non-senescent macrophages to induce TGF-β, pSMAD2/SMAD2, α-SMA, and CTGF in non-senescent fibroblasts. However, the induction levels were lower. Moreover, PLLA-modulated senescent and non-senescent macrophages induced α-SMA and PI3-kinase p85α/AKT in senescent and non-senescent fibroblasts, respectively. COL1A1 and COL3A1 levels in the non-senescent and senescent fibroblasts were also increased by CM from PLLA-treated non-senescent and senescent macrophages, respectively, suggesting that PLLA increased COL1A1 and COL3A1 levels in fibroblasts by modulating macrophages, regardless of senescence.

During skin aging, MMPs cause the destruction of ECM components, such as collagen and elastin fibers [23,24]. Elastin fibers form a unique structure with elastin and fibrillin and maintain the skin’s elasticity. Decreased elastin fiber content leads to decreased skin elasticity [60].

In the present study, PLLA increased the TIMP1 expression level in fibroblasts in the absence of macrophage, regardless of fibroblast senescence. Moreover, PLLA increased TIMP1 levels and decreased MMP2 and MMP3 levels in senescent fibroblasts, probably by modulating senescent macrophages.

In this study, PLLA also caused the synthesis of collagen and elastin fibers in the mouse dermis, regardless of aging. However, the mechanism of action of PLLA in stimulating fibroblasts to synthesize new collagen fibers might be different for senescent and non-senescent fibroblasts. PLLA increased the TGF-β levels in senescent fibroblasts, but not in non-senescent fibroblasts without the involvement of macrophages. PLLA did not alter downstream signaling molecules for TGF-β and CTGF in non-senescent fibroblasts without macrophages. However, the expression levels of COL1A1 and COL3A1 in non-senescent and senescent fibroblasts were increased by PLLA, regardless of macrophage involvement. Moreover, the amount of collagen fibers was increased by PLLA in both young and aged skin. Because collagen synthesis involves complex processes and PLLA induces differential responses in non-senescent and senescent fibroblasts, appropriate experimental models must be selected to evaluate the effects of PLLA on collagen synthesis.

This study has several limitations. First, MMP1 was not evaluated in this study. MMP1, as a main protease, starts collagen fiber destruction, the further fragmentation of which is followed by MMP3 [61,62]. The second limitation was that MMPs were not evaluated in the supernatants of in vitro models. Most MMPs are secreted after translation and promote ECM fragmentation [63]. Thus, MMPs evaluation in the supernatant rather than cells are more clinically relevant model for evaluating PLLA action. Lastly, collagen is formed as precursor form of procollagen and secreted into extracellular space, which further promotes ECM structure formation [64]. Thus, collagen evaluation in the supernatant of the cell is more appropriate to evaluate PLLA action in the ECM.

In conclusion, PLLA increased the IL-4 and IL-13 levels, which led to macrophage polarization toward the M2 subtype. PLLA also increased the TGF-β and IL-10 levels, which led to increased levels of pSMAD2/SMAD2 and CTGF in senescent fibroblasts and aged skin. Moreover, PLLA increased α-SMA and PI3-kinase p85α/AKT, which are involved in differentiation into myofibroblasts and fibroblast proliferation. These changes caused improved collagen synthesis. Finally, PLLA increased TIMP1 and decreased MMPs, leading to decreased ECM destruction in aged skin (Figure 6F).

## Data Availability

Not applicable.

## Supplementary Materials

The following supporting information can be downloaded at: https://www.mdpi.com/article/10.3390/cells12091320/s1, Figure S1: Schematic diagram of the in vitro model used to confirm polarization upon PLLA treatment of H_2_O_2_-induced senescent or non-senescent macrophages; Figure S2: Schematic diagram of the in vitro model used to confirm collagen synthesis in H_2_O_2_-induced senescent or non-senescent fibroblasts following PLLA treatment; Figure S3: Schematic diagram of the in vitro model used to confirm collagen synthesis in fibroblasts exposed to secreted M2 cytokines after PLLA treatment; Figure S4: Schematic diagram of the in vivo model used to confirm collagen synthesis in young and aged mice after PLLA injection; Figure S5: The effect of PLLA on senescence in H_2_O_2_-induced senescent and non-senescent macrophages; Figure S6: PLLA stimulated M2 polarization; Figure S7: Effects of PLLA on senescence in H_2_O_2_-induced senescent and non-senescent fibroblasts; Figure S8: PLLA stimulated collagen synthesis in H_2_O_2_-induced senescent and non-senescent fibroblasts; Figure S9: PLLA reduced senescence in H_2_O_2_-induced senescent fibroblasts exposed to CM from macrophages; Figure S10: PLLA induced collagen synthesis in H_2_O_2_-induced senescent and non-senescent fibroblasts treated with CM from macrophages; Figure S11: PLLA induced collagen synthesis in young and aged skin; Figure S12: PLLA stimulated proliferation in fibroblasts and animal skin via the PI3-kinase p85α/AKT pathway; Table S1: List of antibodies used for ICC, IF, DAB, ELISA and WB.
