# Supplementary material for: Poly-L-Lactic Acid Fillers Improved Dermal Collagen Synthesis by Modulating M2 Macrophage Polarization in Aged Animal Skin
Source: Cells. 2023 May 5;12(9):1320. doi: 10.3390/cells12091320 (PMC10177436; doi:10.3390/cells12091320)
Supplement: Supplementary file 1 [file cells-12-01320-s001.zip › cells-2352684-supplementary.pdf]

cells

MDPI

Article

Poly-L-Lactic Acid Fillers Improved Dermal Collagen Synthesis by Modulating M2 Macrophage Polarization in Aged Animal Skin

Seyeon Oh [1,†], Je Hyuk Lee [2,3,†], Hyoung Moon Kim [3], Sosorburam Batsukh [1,3], Mi Jeong Sung [4], Tae Hwan Lim [4], Myoung Hoon Lee [4], Kuk Hui Son [5,*] and Kyunghee Byun [1,3,6,*]

1. Functional Cellular Networks Laboratory, Lee Gil Ya Cancer and Diabetes Institute, Gachon University, Incheon 21999, Republic of Korea
2. Doctorbom Clinic, Seoul 06614, Republic of Korea
3. Department of Anatomy & Cell Biology, Gachon University College of Medicine, Incheon 21936, Republic of Korea
4. SACCI Bio Co. Seoul 1007, Republic of Korea
5. Department of Thoracic and Cardiovascular Surgery, Gachon University Gil Medical Center, Gachon University, Incheon 21565, Republic of Korea
6. Department of Health Sciences and Technology, Gachon Advanced Institute for Health & Sciences and Technology (GAIHST), Gachon University, Incheon 21999, Republic of Korea

* Correspondence: dr632@gilhospital.com (K.H.S.); khbyun1@gachon.ac.kr (K.B.); Tel.: +82-32-460-3666 (K.H.S.); +82-32-899-6511 (K.B.)

† These authors contributed equally to this study.

Citation: Oh, S.; Lee, J.H.; Kim, H.M.; Batsukh, S.; Sung, M.J.; Lim, T.H.; Lee, M.H.; Son, K.H.; Byun, K. Poly-L-Lactic Acid Fillers Improved Dermal Collagen Synthesis by Modulating M2 Macrophage Polarization in Aged Animal Skin. *Cells* **2023**, *12*, 1320. https://doi.org/10.3390/cells12091320

Academic Editor: Nicoletta Gagliano

Received: 2 April 2023
Revised: 1 May 2023
Accepted: 2 May 2023
Published: 5 May 2023

Copyright: © 2023 by the authors. Licensee MDPI, Basel, Switzerland. This article is an open access article distributed under the terms and conditions of the Creative Commons Attribution (CC BY) license (https://creativecommons.org/licenses/by/4.0/).

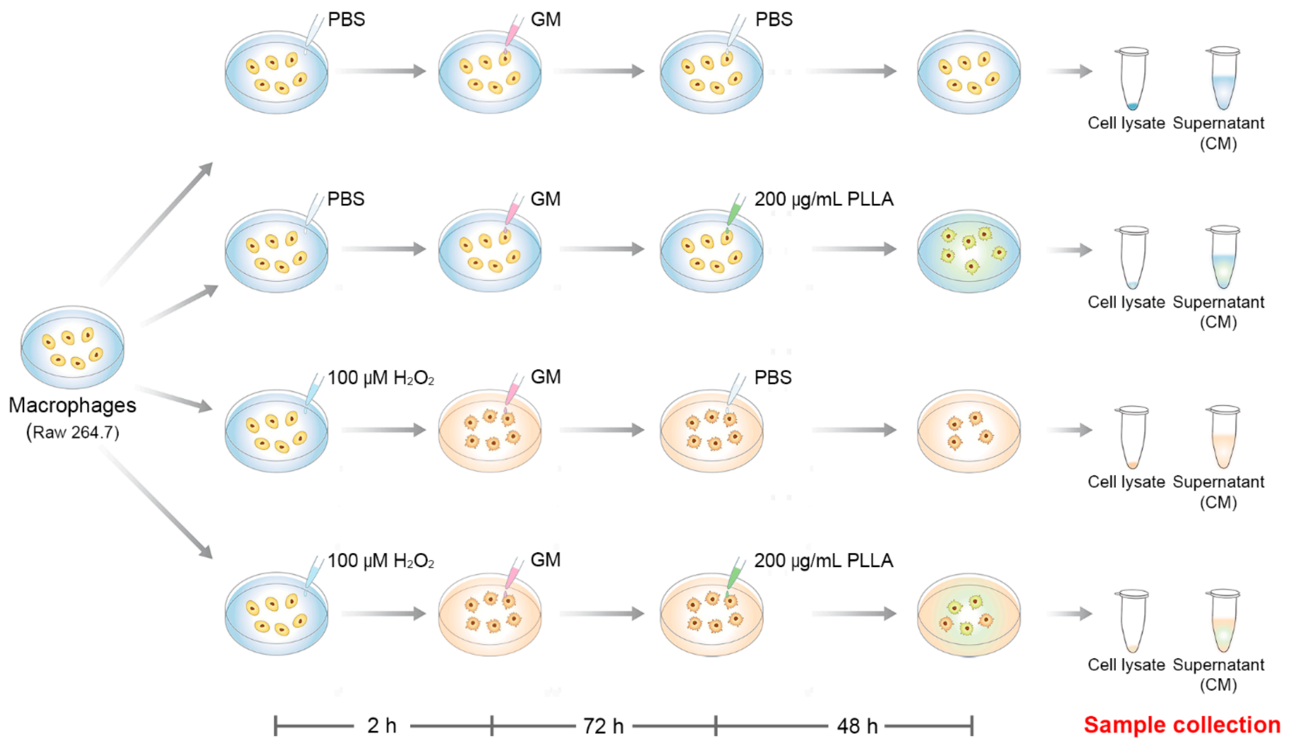

Figure S1. Schematic diagram of the *in vitro* model used to confirm polarization upon PLLA treatment of H_2O_2-induced senescent or non-senescent macrophages. The macrophages (Raw 264.7) were treated with PBS or H_2O_2 (100 µM) for 2 h, which was replaced with GM for 72 h after washing with DPBS. After washing with DPBS, PBS or PLLA (200 µg/mL) was mixed with growth media, and the cells were cultured for 48 h. After the incubation, the supernatant (CM) was collected, and cell lysates were prepared from the treated cells. CM, conditioned medium; DPBS, Dulbecco's phosphate-buffered-saline; GM, growth medium; h, hours; H_2O_2, hydrogen peroxide; µg, microgram; mL, milliliter; µM, micromolar; PBS, phosphate-buffered-saline; PLLA, poly L-lactic acid.

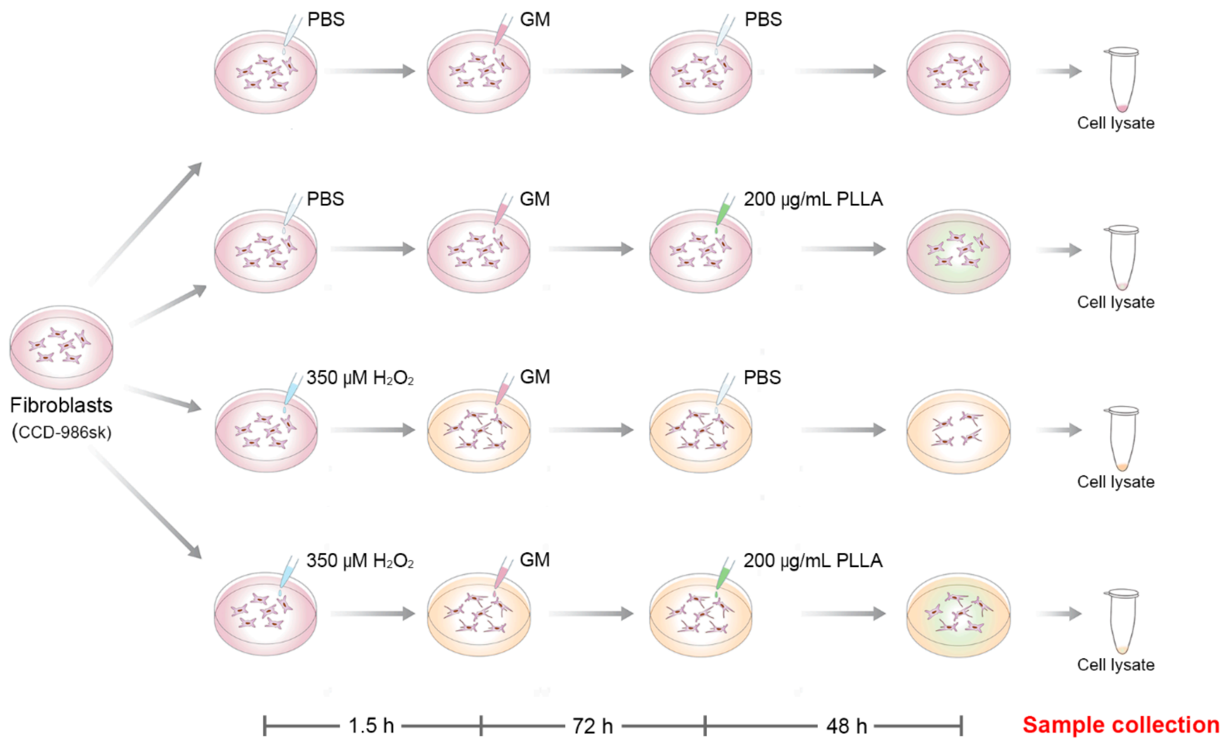

Figure S2. Schematic diagram of the *in vitro* model used to confirm collagen synthesis in H_2O_2-induced senescent or non-senescent fibroblasts following PLLA treatment. Fibroblasts (CCD-986sk) were treated with PBS or H_2O_2 (350 µM) for 1.5 h and replaced with GM for 72 h after washing with DPBS. The cells were then cultured with PBS or PLLA (200 µg/mL) in GM for 48 h. After the incubation, cell lysates were prepared from the treated cells. DPBS, Dulbecco's phosphate-buffered-saline; GM, growth medium; h, hours; H_2O_2, hydrogen peroxide; µg, microgram; mL, milliliter; µM, micromolar; PBS, phosphate -buffered-saline; PLLA, poly L-lactic acid.

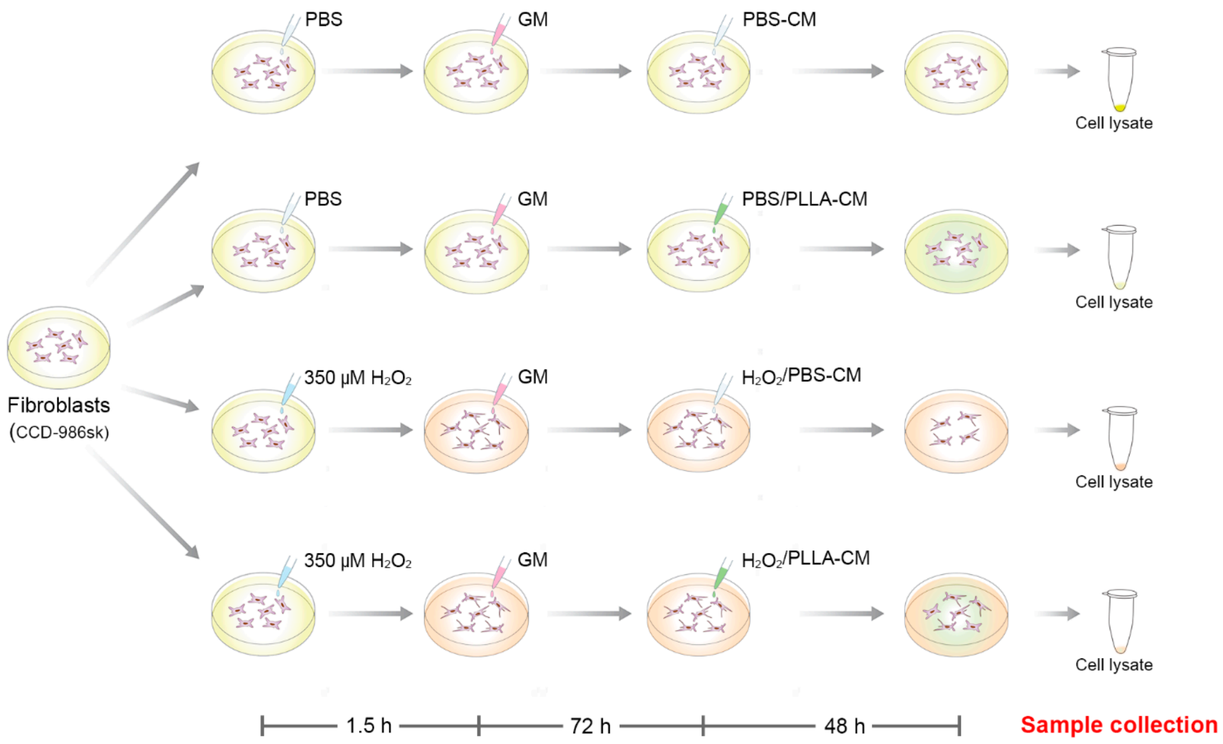

Figure S3. Schematic diagram of the *in vitro* model used to confirm collagen synthesis in fibroblasts exposed to secreted M2 cytokines after PLLA treatment. Macrophages were treated with H_2O_2 (100 μM for 2 h) to induce senescence or PBS as a control. Both sets of macrophages were then treated with PBS or PLLA (200 μg/mL) for 48 h. As mentioned in Figure S1, after 48 h, the CM was collected. Senescence-induced fibroblasts were treated with the CM for 48 h. After the incubation, cell lysates were prepared from the treated fibroblasts. CM, conditioned medium; DPBS, Dulbecco's phosphate-buffered-saline; GM, growth medium; h, hours; H_2O_2, hydrogen peroxide; μg, microgram; mL, milliliter; μM, micromolar; PBS, phosphate-buffered-saline; PLLA, poly L-lactic acid.

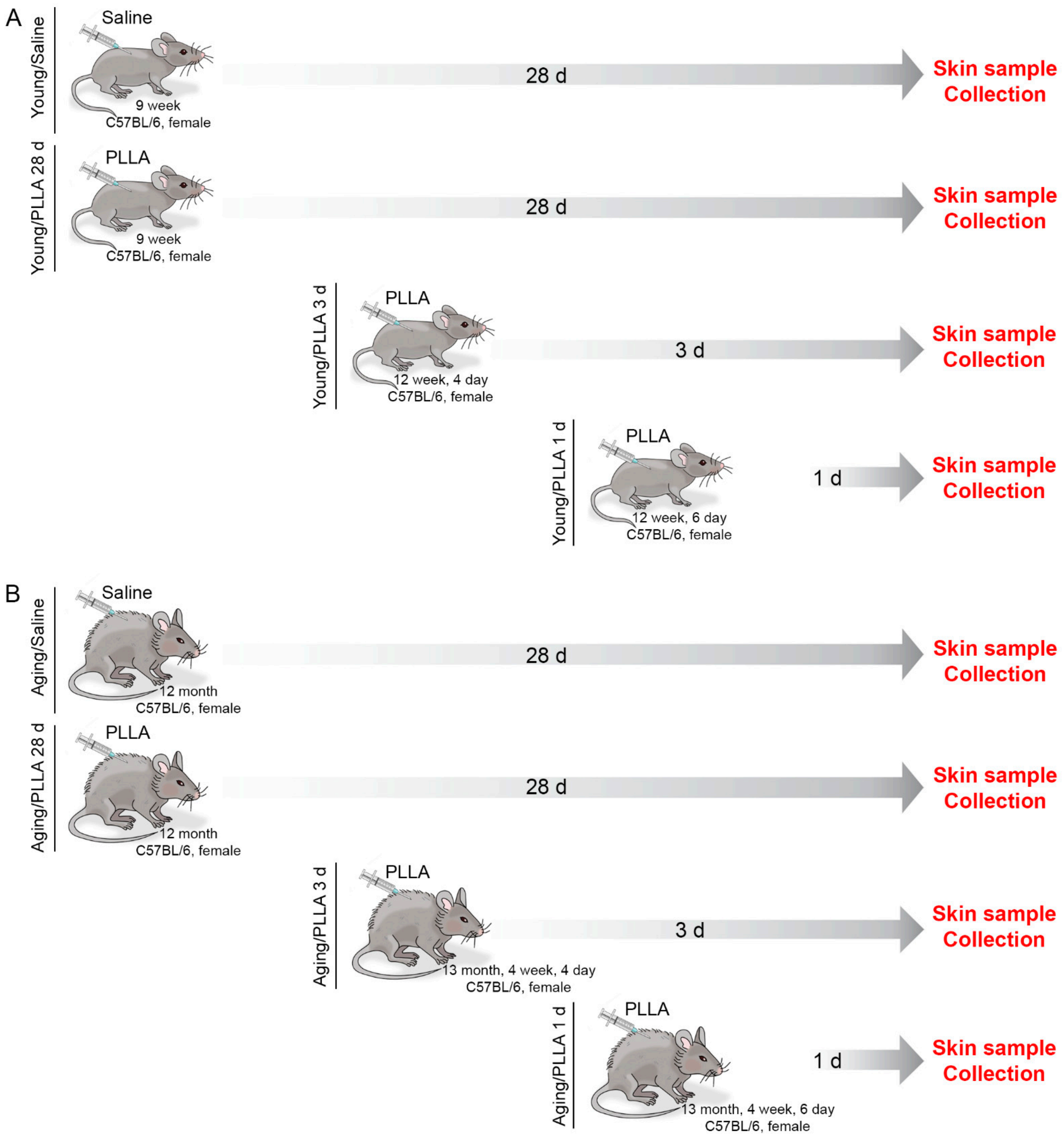

Figure S4. Schematic diagram of the *in vivo* model used to confirm collagen synthesis in young and aged mice after PLLA injection. Saline or 10 mg/mL PLLA (100 µL) was injected into the dermis of each mouse back in five different areas. Skin tissues were sacrificed 1, 3, and 28 days later. The animal experiment was designed to adjust the age of the mice to 13 weeks (Young mice) and 14 months (Aging mice), respectively, when the skin tissue was harvested. d, days; PLLA, poly L-lactic acid.

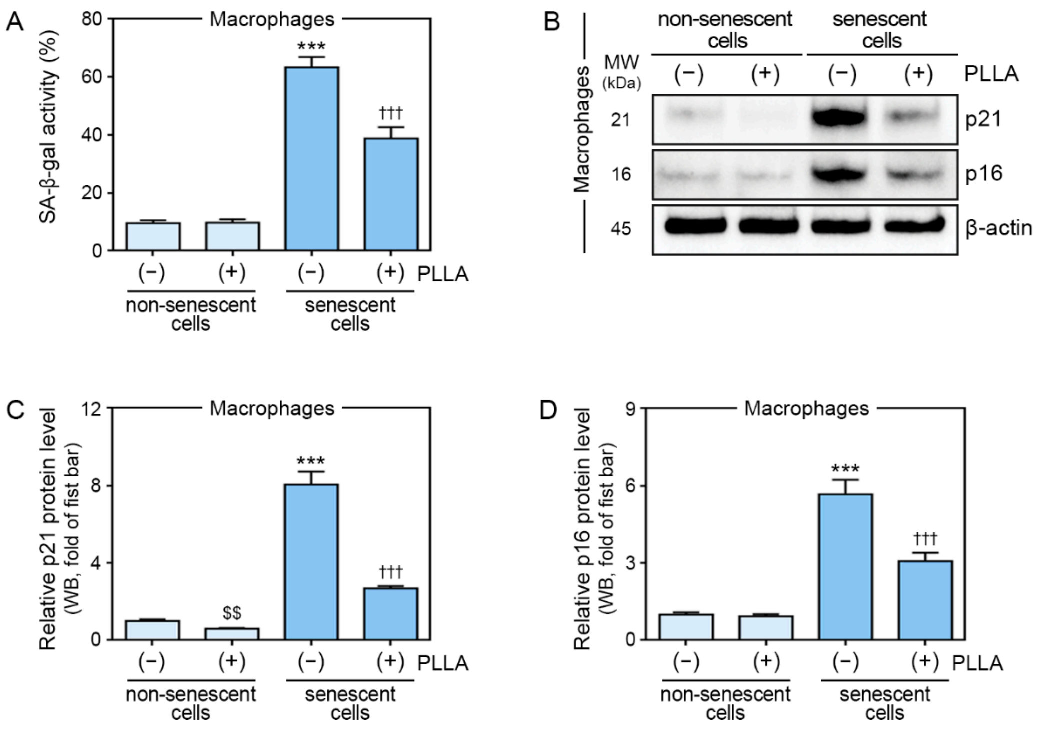

Figure S5. The effect of PLLA on senescence in H_2O_2-induced senescent and non-senescent macrophages. H_2O_2-induced senescent and non-senescent macrophages were treated with PBS (first or third bar) or PLLA (second or fourth bar). (**A**) SA-β-gal activity was measured in H_2O_2-induced senescent and non-senescent macrophages. (**B**) The expression of p21 and p16 (senescence markers) in H_2O_2-induced senescent and non-senescent macrophages was analyzed by western blotting. (**C, D**) Graph quantifying the data in (**B**). Data are presented as the mean ± SD (n = 3/group). $$, $p < 0.01$, first bar vs. second bar; ***, $p < 0.001$, first bar vs. third bar; †††, $p < 0.001$, third bar vs. fourth bar. H_2O_2, hydrogen peroxide; kDa, kilodalton; MW, molecular weight; PBS, phosphate buffered saline; PLLA, poly-L-lactic acid; SA-β-gal, senescence-associated beta-galactosidase; SD, standard deviation; WB, western blotting.

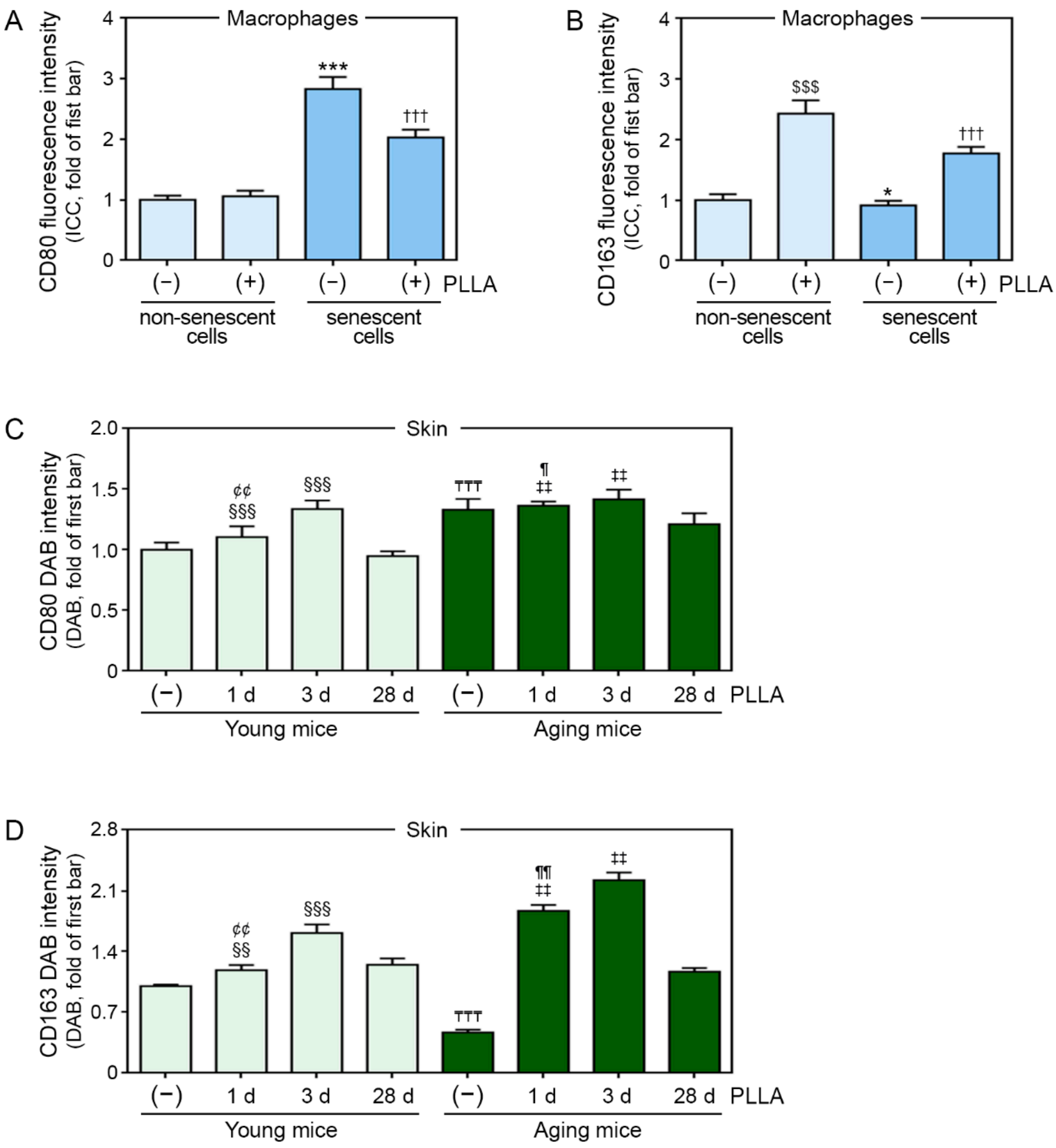

Figure S6. PLLA stimulated M2 polarization. H_2O_2-induced senescent and non-senescent macrophages were treated with PBS (first or third bar) or PLLA (second or fourth bar). (**A, B**) The expression of CD80 (M1 marker) and CD163 (M2 marker) in H_2O_2-induced senescent and non-senescent macrophages was analyzed using immunocytochemistry. Graph quantifying the data in Figure 2C. Young or aging mice were injected saline or PLLA and skin samples were collected after 1 (2nd or 6th bar), 3 (3rd or 7th bar), and 28 days (1st, 4th, 5th, or 8th bar). (**C, D**) The expression of CD80 and CD163 in young and aged skin was analyzed using DAB staining. Graph quantifying the data in Figure 2F. Data are presented as the mean ± SD (n = 3/group). $$$, $p < 0.001$, first bar vs. second bar in (**A,B**); * and ***, $p < 0.05$ and $p < 0.001$, first bar vs. third bar in (**A,B**); †††, $p < 0.001$, third bar vs. fourth bar in (**A,B**); ⊤⊤⊤, $p < 0.001$, first bar vs. fifth bar in (**C,D**); ¢¢, $p < 0.01$, third bar vs. second bar in (**C,D**); §§ and §§§, $p < 0.01$ and $p < 0.001$, fourth bar vs. second or third bar in (**C,D**); ¶ and ¶¶, $p < 0.05$ and $p < 0.01$, seventh bar vs. sixth bar in (**C,D**); ‡‡, $p < 0.01$, eighth bar vs. sixth or seventh bar in (**C,D**). CD80, cluster of differentiation 80; CD163, cluster of differentiation 163; d, days; DAB, 3,3-diaminobenzidine; H_2O_2, hydrogen peroxide; ICC, immunocytochemistry; PBS, phosphate buffered saline; DAB, 3,3'-diaminobenzidine; PLLA, poly-L-lactic acid; SD, standard deviation.

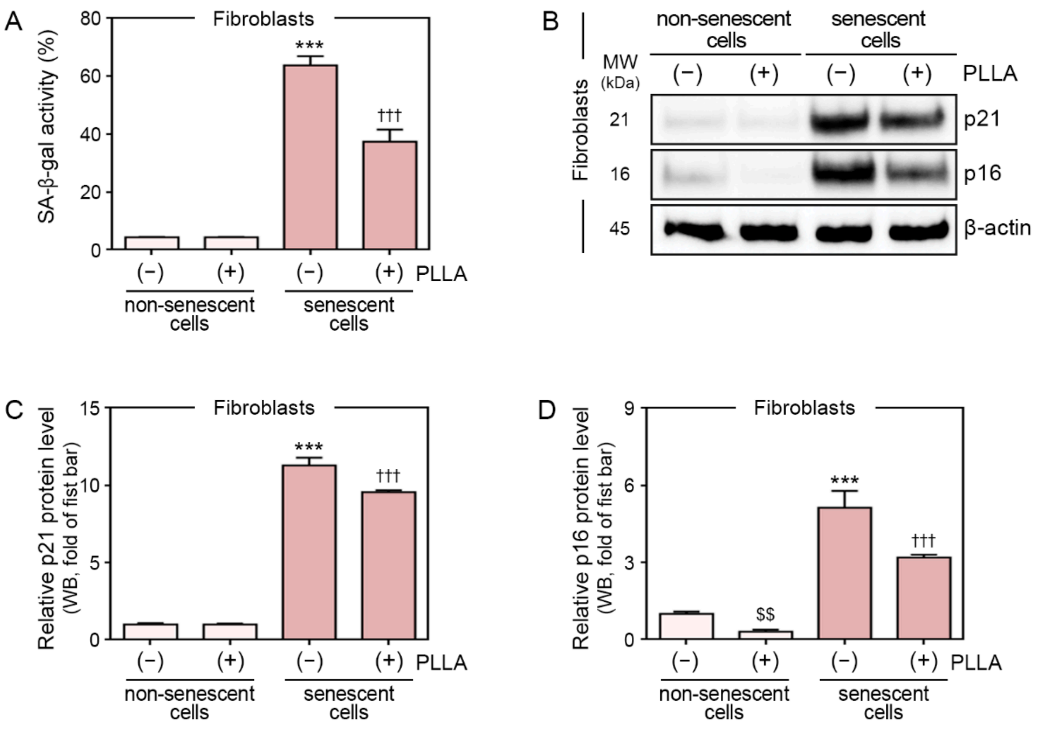

Figure S7. Effects of PLLA on senescence in H_2O_2-induced senescent and non-senescent fibroblasts. H_2O_2-induced senescent and non-senescent fibroblasts were treated with PBS (first or third bar) or PLLA (second or fourth bar). (**A**) SA-β-gal activity was measured in H_2O_2-induced senescent and non-senescent fibroblasts. (**B**) The expression of p21 and p16 (senescence markers) in H_2O_2-induced senescent and non-senescent fibroblasts was analyzed by western blotting. (**C, D**) Graph quantifying the data in (**B**). Data are presented as the mean ± SD (n = 3/group). $$, $p < 0.01$, first bar vs. second bar; ***, $p < 0.001$, first bar vs. third bar; †††, $p < 0.001$, third bar vs. fourth bar. H_2O_2, hydrogen peroxide; kDa, kilodalton; MW, molecular weight; PBS, phosphate buffered saline; PLLA, poly-L-lactic acid; SA-β-gal, senescence-associated beta-galactosidase; SD, standard deviation; WB, western blotting.

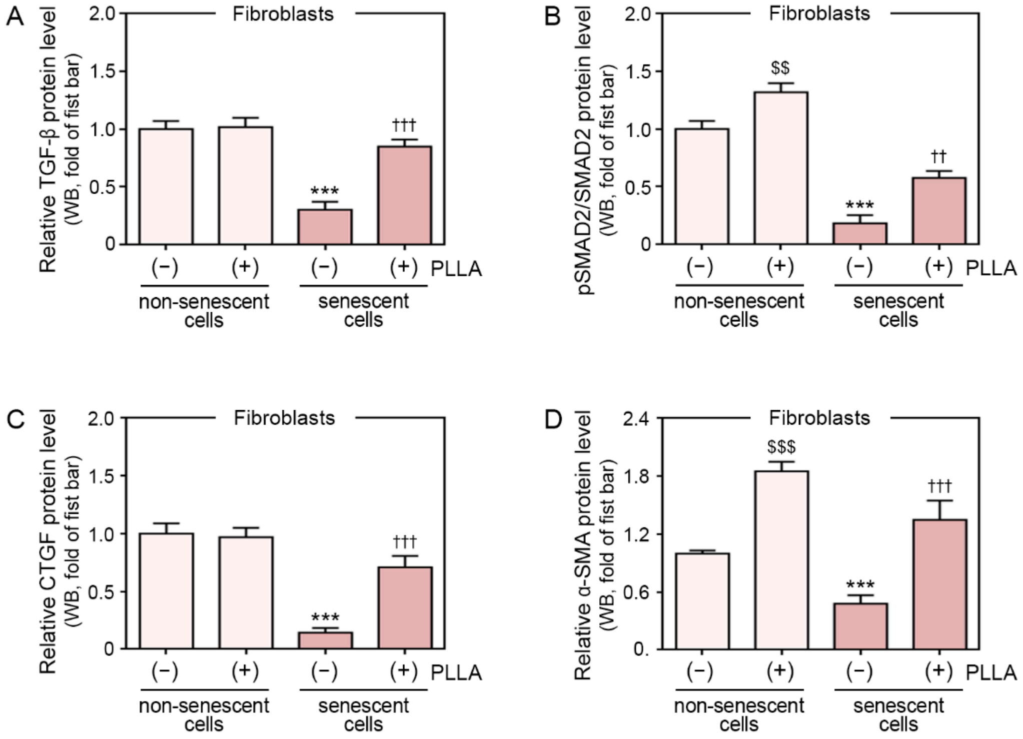

Figure S8. PLLA stimulated collagen synthesis in H_2O_2-induced senescent and non-senescent fibroblasts. H_2O_2-induced senescent and non-senescent fibroblasts were treated with PBS (first or third bar) or PLLA (second or fourth bar). (**A–D**) TGF-β, pSMAD2/SMAD2, CTGF and α-SMA levels in H_2O_2-induced senescent and non-senescent fibroblasts were analyzed by western blotting. Graph quantifying the data in Figure 3A. Data are presented as the mean ± SD (n = 3/group). $$ and $$$, p < 0.01 and p < 0.001, first bar vs. second bar; ***, p < 0.01 and p < 0.001, first bar vs. third bar; †† and †††, p < 0.01 and p < 0.001, third bar vs. fourth bar. α-SMA, alpha-smooth muscle actin; CTGF, connective tissue growth factor; H_2O_2, hydrogen peroxide; PBS, phosphate buffered saline; PLLA, poly-L-lactic acid; SD, standard deviation; TGF-β, transforming growth factor-beta; WB, western blotting.

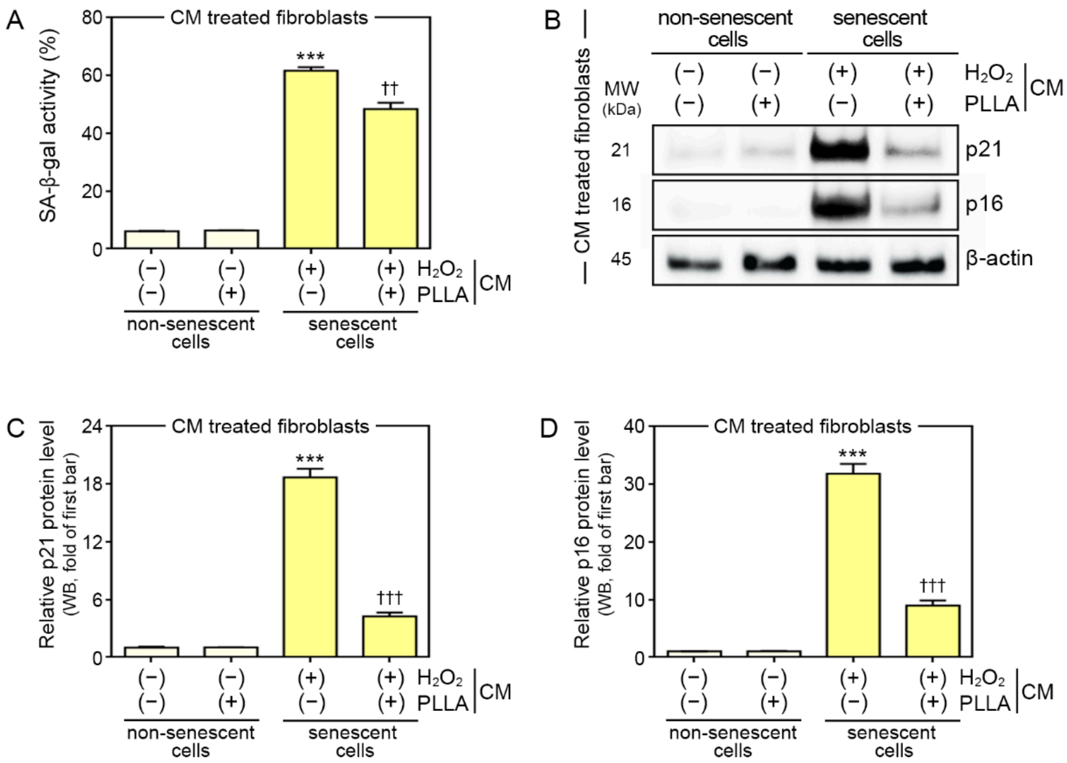

Figure S9. PLLA reduced senescence in H_2O_2-induced senescent fibroblasts exposed to CM from macrophages. Senescent or non-senescent fibroblasts were treated with CM from macrophages treated with PBS (first bar), PBS/PLLA (second bar), H_2O_2/PBS (third bar), or H_2O_2/PLLA (fourth bar). (**A**) SA-β-gal activity was measured in H_2O_2-induced senescent and non-senescent fibroblasts. (**B**) The expression of p21 and p16 (senescence markers) in H_2O_2-induced senescent and non-senescent fibroblasts was analyzed by western blotting. (**C, D**) Graph quantifying the data in (**B**). Data are presented as the mean ± SD (n = 3/group). ***, $p < 0.001$, first bar vs. third bar; †† and †††, $p < 0.01$ and $p < 0.001$, third bar vs. fourth bar. CM, conditioned medium; H_2O_2, hydrogen peroxide; kDa, kilodalton; MW, molecular weight; PBS, phosphate buffered saline; PLLA, poly-L-lactic acid; SA-β-gal, senescence-associated beta-galactosidase; SD, standard deviation; WB, western blotting.

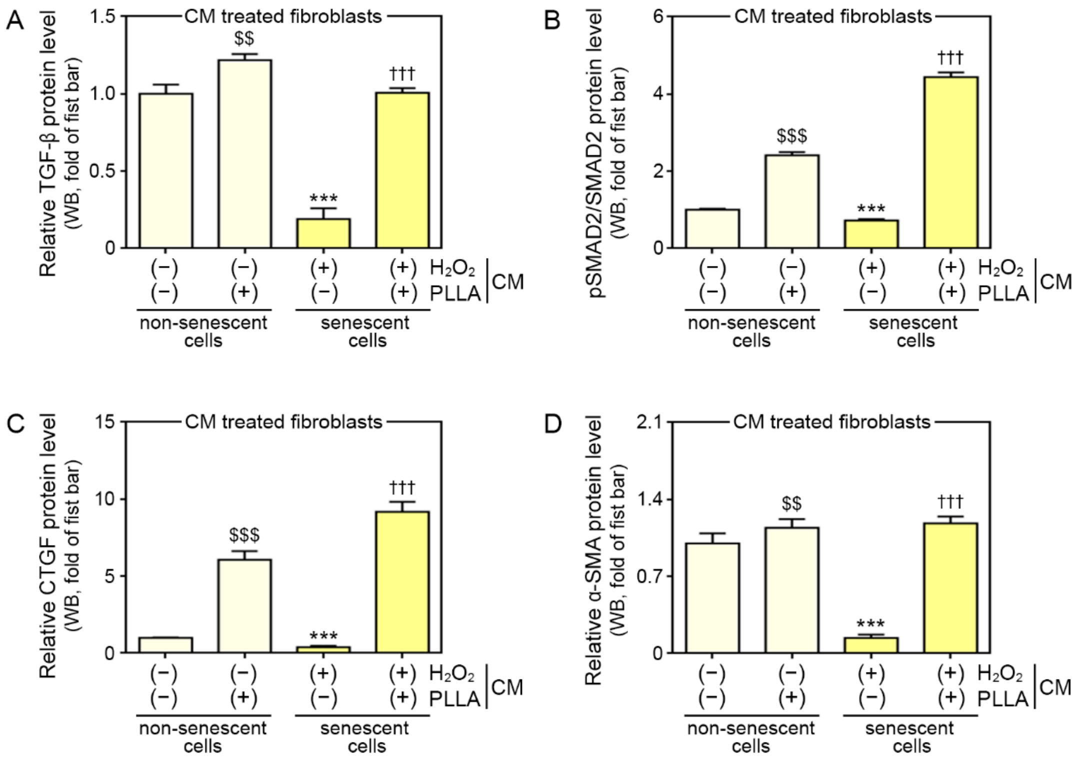

Figure S10. PLLA induced collagen synthesis in H_2O_2-induced senescent and non-senescent fibroblasts treated with CM from macrophages. Senescent or non-senescent fibroblasts were treated with CM from macrophages treated with PBS (first bar), PBS/PLLA (second bar), H_2O_2/PBS (third bar), or H_2O_2/PLLA (fourth bar). (**A–D**) TGF-β, pSMAD2/SMAD2, CTGF, and α-SMA levels in H_2O_2-induced senescent and non-senescent fibroblasts were analyzed by western blots. Graph quantifying the data in Figure 3D. Data are presented as the mean ± SD (n = 3/group). $$ and $$$, $p < 0.01$ and $p < 0.001$, first bar vs. second bar; ***, $p < 0.001$, first bar vs. third bar; †††, $p < 0.001$, third bar vs. fourth bar. α-SMA, alpha-smooth muscle actin; CM, conditioned medium; CTGF, connective tissue growth factor; H_2O_2, hydrogen peroxide; PBS, phosphate buffered saline; PLLA, poly-L-lactic acid; SD, standard deviation; TGF-β, transforming growth factor-beta; WB, western blotting.

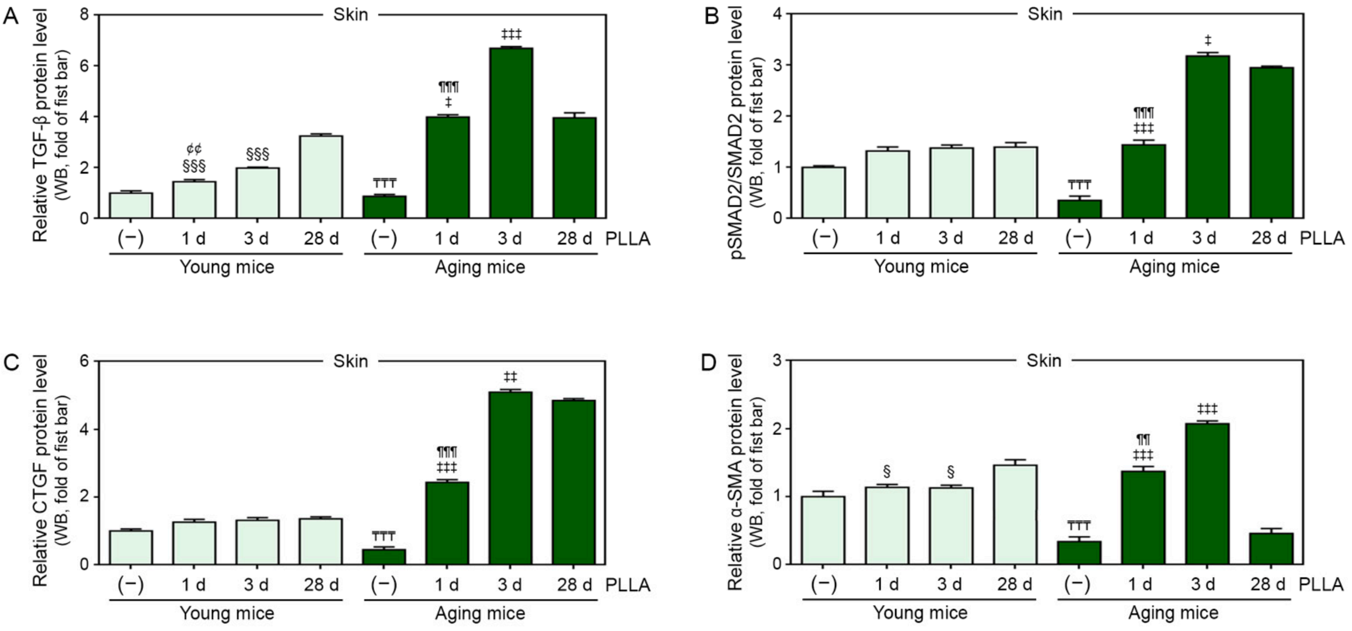

Figure S11. PLLA induced collagen synthesis in young and aged skin. PLLA was injected into the dermis of young and aged skin, and the skin was collected after 1 (2nd or 6th bar), 3 (3rd or 7th bar), or 28 (1st, 4th, 5th, or 8th bar) days. (**A–D**) TGF-β, pSMAD2/SMAD2, CTGF and α-SMA levels in young and aged skin were analyzed by western blotting. Graph quantifying the data in Figure 3G. Data are presented as the mean ± SD (n = 3/group). ⊤⊤⊤, $p < 0.001$, first bar vs. fifth bar; ¢¢, $p < 0.01$, third bar vs. second bar; § and §§§, $p < 0.05$ and $p < 0.001$, fourth bar vs. second or third bar; ¶¶ and ¶¶¶, $p < 0.01$ and $p < 0.001$, seventh bar vs. sixth bar; ‡, ‡‡ and ‡‡‡, $p < 0.05$, $p < 0.01$ and $p < 0.001$, eighth bar vs. sixth or seventh bar. α-SMA, alpha-smooth muscle actin; CTGF, connective tissue growth factor; d, days; PLLA, poly-L-lactic acid; SD, standard deviation; TGF-β, transforming growth factor-beta; WB, western blotting.

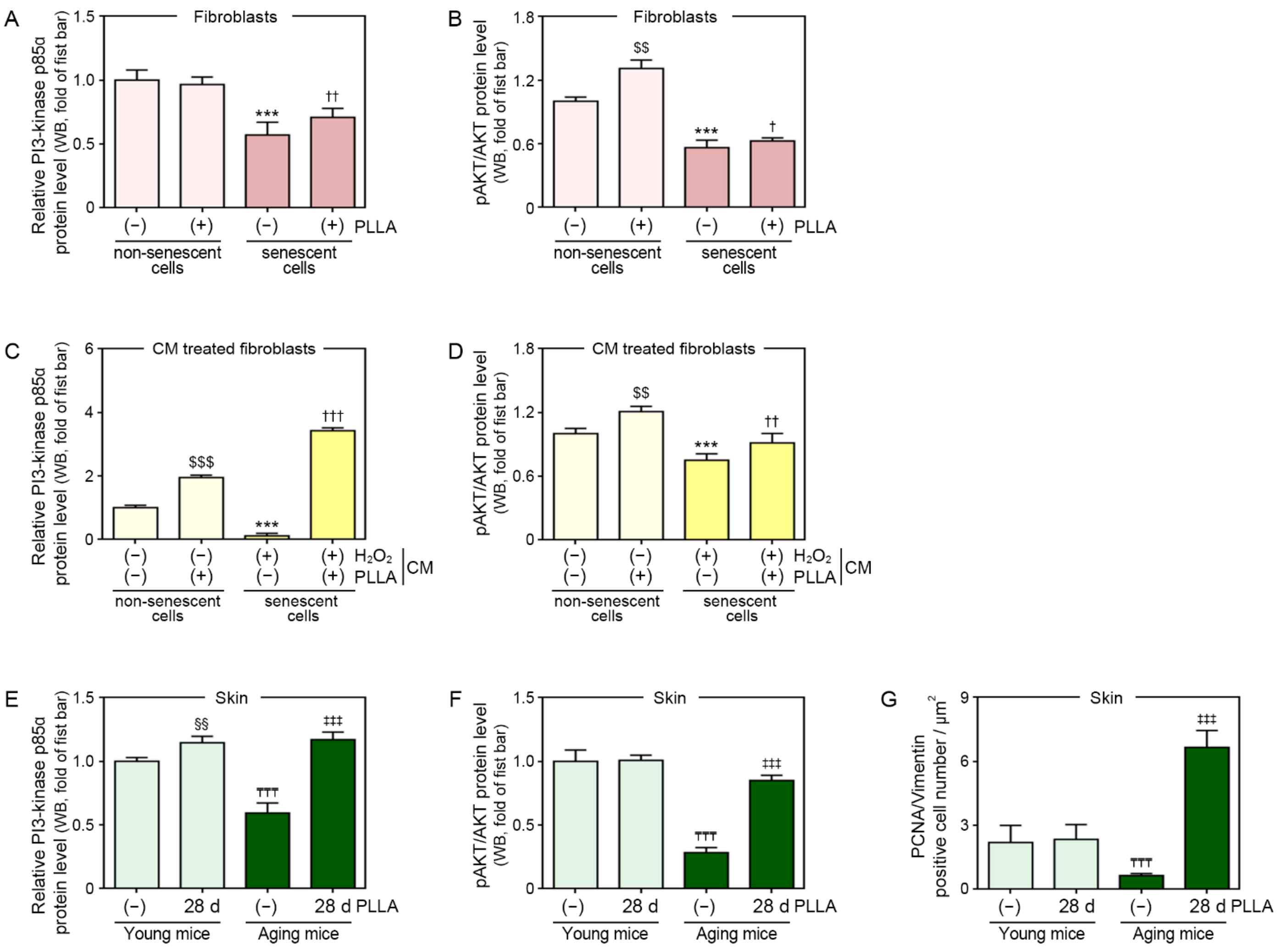

Figure S12. PLLA stimulated proliferation in fibroblasts and animal skin via the PI3-kinase p85α/AKT pathway. H_2O_2-induced senescent and non-senescent fibroblasts were treated with PBS (first or third bar) or PLLA (second or fourth bar). (**A, B**) PI3-kinase p85α, pAKT/AKT levels in H_2O_2-induced senescent and non-senescent fibroblasts were analyzed by western blotting. Graph quantifying the data in Figure 4A. Senescent or non-senescent fibroblasts were treated with CM from macrophages treated with PBS (first bar), PBS/PLLA (second bar), H_2O_2/PBS (third bar), or H_2O_2/PLLA (fourth bar). (**C, D**) PI3-kinase p85α, pAKT/AKT levels in H_2O_2-induced senescent and non-senescent fibroblasts were analyzed by western blotting. Graph quantifying the data in Figure 4C. PLLA was injected into the dermis of young and aged skin, and the skin was collected after 1 (2nd or 6th bar), 3 (3rd or 7th bar), or 28 (1st, 4th, 5th, or 8th bar) days. (**E, F**) PI3-kinase p85α, pAKT/AKT levels in young and aged skin were analyzed by western blotting. Graph quantifying the data in Figure 4E. (**G**) The co-stained cell number of vimentin (fibroblast marker) and PCNA (proliferation marker) in young and aged skin was analyzed using immunofluorescence. Graph quantifying the data in Figure 4F. Data are presented as the mean ± SD (n = 3/group). $$ and $$$, $p < 0.01$ and $p < 0.001$, first bar vs. second bar in (**A–D**); ***, $p < 0.001$, first bar vs. third bar in (**A–D**); †, †† and †††, $p < 0.05$, $p < 0.01$ and $p < 0.001$, third bar vs. fourth bar in (**A–D**); §§, $p < 0.01$, first bar vs. second bar in (**E–G**); ⸆⸆⸆, $p < 0.001$, first bar vs. third bar in (**E–G**); ‡‡‡, $p < 0.001$, third bar vs. fourth bar in (**E–G**). CM, conditioned medium; H_2O_2, hydrogen peroxide; phosphate buffered saline; pAKT, phosphorylated AKT; PCNA, proliferating cell nuclear antigen; PLLA, poly-L-lactic acid; SD, standard deviation; WB, western blotting.

Table S1. List of antibodies used for ICC, IF, DAB, ELISA and WB.

Antibody name	Company	Catalog number	Dilution ratio				
			ICC	IF	DAB	ELISA	WB
CD80	Abclonal	A16039	1:200		1:100		
CD163	Santa Cruz Biotechnology	sc-58965	1:50		1:50		
IL-4	CUSABIO	CSB-PA011659YA01HU				1:500	
IL-13	FineTest	FNab10489				1:500	
TGF-β	Abcam	ab64715					1:2,000
SMAD2	Cell signaling Technology	8685s					1:1,000
pSMAD2	Cell signaling Technology	8828s					1:1,000
α-SMA	Invitrogen Fisher Scientific	14-9760-82					1:2,000
CTGF	Santa Cruz Biotechnology	sc-373936					1:500
COL1A1	Santa Cruz Biotechnology	sc-293182				1:30	
COL3A1	Bioss	bs-0549R				1:1,000	
PI3-kinase p85α	Santa Cruz Biotechnology	sc-376112					1:200
AKT	BD Biosciences	610860					1:1,000
pAKT	Abcam	ab81283					1:1,000
Vimentin	Santa Cruz Biotechnology	sc-373717		1:50			
PCNA	Abcam	ab18197		1:100			
IL-10	Santa Cruz Biotechnology	sc-365858					1:200
TIMP1	St John's Laboratory Ltd	STJ96023					1:500
MMP2	LSBio	LS-C352523					1:500
MMP3	CUSABIO	CSB-PA07449A0Rb					1:1,000
β-actin	Cell signaling Technology	4967s					1:1,000

AKT, protein kinase B; α-SMA, alpha-smooth muscle actin; β-actin, beta-actin; β-actin, beta-actin; CD80, cluster of differentiation 80; CD163, cluster of differentiation 163; COL1A1, collagen type 1A1; COLA3A1, collagen type 3A1; CTGF, connective tissue growth factor; DAB, 3,3'-diaminobenzidine; ELISA, enzyme-linked immunosorbent assay; ICC, immunocytochemistry; IF, immunofluorescence; IL-4, interleukin-4; IL-10, interleukin-10; IL-13, interleukin-13; MMP2, matrix metalloproteinase 2; MMP3, matrix metalloproteinase 3; pAKT, phosphorylated AKT; PCNA, proliferating cell nuclear antigen; PI3-kinase p85α, phosphatidylinositol 3-kinase p85α; pSMAD2, phosphorylated SMAD2; TGF-β, transforming growth factor-beta; TIMP1, tissue inhibitor of metalloproteinase 1; WB, western blotting.
